# Feasibility cluster randomised controlled trial evaluating a theory-driven group-based complex intervention versus usual physiotherapy to support self-management of osteoarthritis and low back pain (SOLAS)

**DOI:** 10.1186/s13063-020-04671-x

**Published:** 2020-09-23

**Authors:** Deirdre A. Hurley, Isabelle Jeffares, Amanda M. Hall, Alison Keogh, Elaine Toomey, Danielle McArdle, Suzanne M. McDonough, Suzanne Guerin, Ricardo Segurado, James Matthews

**Affiliations:** 1 School of Public Health, Physiotherapy and Sports Science, University College Dublin, Room A302, Health Sciences Centre, Belfield, Dublin 4, Ireland; 2 Division of Population Health Sciences, Royal College of Surgeons in Ireland, St Stephen’s Green, Dublin 2, Ireland; 3 Faculty of Medicine, Memorial University, St Johns, Newfoundland Canada; 4 Health Behaviour Change Research Group, School of Psychology, National University of Ireland, Galway, Ireland; 5 School of Physiotherapy, Royal College of Surgeons in Ireland, St Stephen’s Green, Dublin 2, Ireland; 6 School of Psychology, University College Dublin, Dublin, Ireland

**Keywords:** Complex group intervention, Feasibility cluster randomised controlled trial, Self-management, Behaviour change intervention, Qualitative methods, Intervention mapping, Osteoarthritis, Low back pain, Physiotherapists, Primary care

## Abstract

**Background:**

The self-management of osteoarthritis (OA) and low back pain (LBP) through activity and skills (SOLAS) theory-driven group-based complex intervention was developed primarily for the evaluation of its acceptability to patients and physiotherapists and the feasibility of trial procedures, to inform the potential for a definitive trial.

**Methods:**

This assessor-blinded multicentre two-arm parallel cluster randomised controlled feasibility trial compared the SOLAS intervention to usual individual physiotherapy (UP; pragmatic control group). Patients with OA of the hip, knee, lumbar spine and/or chronic LBP were recruited in primary care physiotherapy clinics (i.e. clusters) in Dublin, Ireland, between September 2014 and November 2015. The primary feasibility objectives were evaluated using quantitative methods and individual telephone interviews with purposive samples of participants and physiotherapists. A range of secondary outcomes were collected at baseline, 6 weeks (behaviour change only), 2 months and 6 months to explore the preliminary effects of the intervention. Analysis was by intention-to-treat according to participants’ cluster allocation and involved descriptive analysis of the quantitative data and inductive thematic analysis of the qualitative interviews. A linear mixed model was used to contrast change over time in participant secondary outcomes between treatment arms, while adjusting for study waves and clusters.

**Results:**

Fourteen clusters were recruited (7 per trial arm), each cluster participated in two waves of recruitment, with the average cluster size below the target of six participants (intervention: mean (SD) = 4.92 (1.31), range 2–7; UP: mean (SD) = 5.08 (2.43), range 1–9). One hundred twenty participants (83.3% of *n* = 144 expected) were recruited (intervention *n* = 59; UP *n* = 61), with follow-up data obtained from 80.8% (*n* = 97) at 6 weeks, 84.2% (*n* = 101) at 2 months and 71.7% (*n* = 86) at 6 months. Most participants received treatment as allocated (intervention *n* = 49; UP *n* = 54). The qualitative interviews (12 participants; 10 physiotherapists (PTs) found the intervention and trial procedures acceptable and appropriate, with minimal feasible adaptations required. Linear mixed methods showed improvements in most secondary outcomes at 2 and 6 months with small between-group effects.

**Conclusions:**

While the SOLAS intervention and trial procedures were acceptable to participants and PTs, the recruitment of enough participants is the biggest obstacle to a definitive trial.

**Trial registration:**

ISRCTN ISRCTN49875385. Registered on 26 March 2014.

## Introduction

**Fig. 1:**
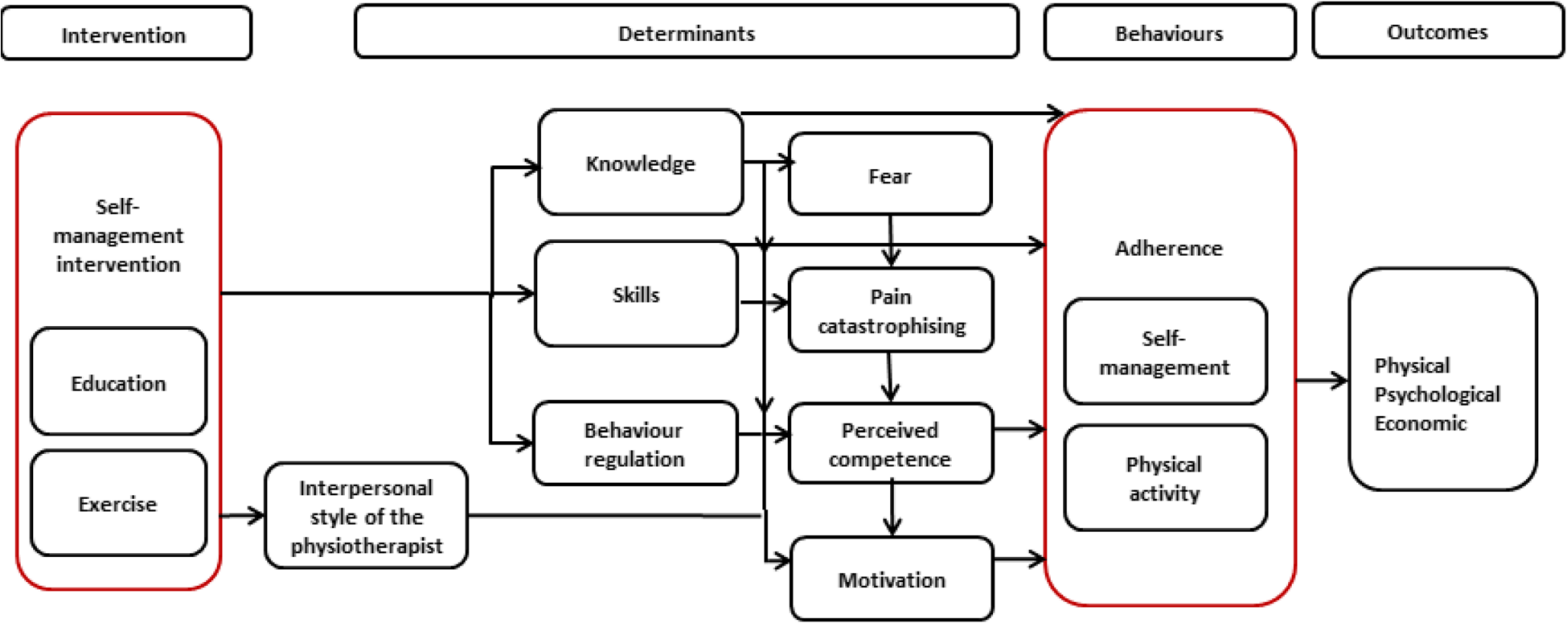
Process model of behaviour change in SOLAS intervention

The successful implementation of a standardised, evidence-based group programme to support self-management (SM) for people with chronic musculoskeletal pain is a priority for primary care physiotherapy (PT) in Ireland [1]. While international clinical guidelines endorse self-management, exercise and physical activity for osteoarthritis (OA) and low back pain (LBP) [2–6], the evidence for the effectiveness of existing programmes is weak, of low quality [7–9] and rarely underpinned by behaviour change theory [10, 11]. Additional local barriers to implementation of these guidelines in Ireland include the variable quality of the primary care health system infrastructure and physiotherapy staffing levels, resulting in most patients with OA and LBP receiving a plethora of multi-modal individual physiotherapy approaches from staff with varying levels of expertise in chronic musculoskeletal pain [1]. We developed the self-management of OA and chronic LBP through activity and skills (SOLAS) complex intervention, by adapting an existing intervention (Facilitating Activity and Self-management in Arthritis, FASA) [12] through an intervention mapping (IM) process [13]. FASA is a version of the efficacious and cost-effective ESCAPE-knee pain intervention [14, 15] designed for patients with OA of the hip, knee and lumbar spine with proven acceptability in the UK health system [16], and hence, it was considered a credible prototype for adaptation. The IM process included a needs assessment involving literature reviews, interviews with patients with OA and LBP and primary care physiotherapists, evaluation of existing primary care physiotherapy resources to provide a standardised group programme, and a consensus building workshop with physiotherapy stakeholders to define the SOLAS intervention programme goals, underpinning behaviour change theory and required adaptations to FASA to address the needs of patients, the health service and the evidence [13]. The resultant SOLAS intervention comprises six weekly sessions of 90-min group education and exercise designed for people aged at least 45 years with OA of the hip, knee and/or lumbar spine and those aged at least 30 years with chronic LBP. This contrasts with the FASA and ESCAPE interventions of 12 twice weekly sessions of 60 min intended for adults aged over 50 years with OA only. SOLAS is also underpinned by self-determination theory (SDT), which proposes that people have basic psychological needs for autonomy, competence and relatedness, which if satisfied, for example, by the needs supportive communication style of a physiotherapist will increase individuals’ autonomous motivation and engagement in our target health behaviours of increased physical activity (PA) and use of self-management strategies. SOLAS also targets other selected determinants of SM behaviour, including fear and pain catastrophising in LBP patients [5, 17–19] identified from our needs assessment, via 31 evidence-based behaviour change techniques (BCTs), defined within the intervention mapping process, as illustrated in Fig. 1. Consequently, SOLAS is the first theory-driven, group-based intervention designed for a mixed group of people with OA and/or chronic LBP (CLBP) that was informed by the needs of intervention providers and patients to increase its potential for implementation. Therefore, as endorsed within the UK Medical Research Council guidelines, the credibility, acceptability and feasibility of this intervention warrants investigation prior to testing in a definitive trial [20].

## Aims and objectives

The aim of this cluster feasibility trial [ISRCTN49875385] was to evaluate the feasibility of providing the SOLAS intervention [experimental group] within a diverse range of primary care PT settings for patients with OA hip/ knee, lumbar spine and/or CLBP compared to usual individual physiotherapy (UP), which served as the pragmatic control group in order to inform its appropriateness for testing in a future definitive trial.

Based on key areas of focus for feasibility studies [21–24], our primary objectives were (1) to assess the acceptability, demand and necessary adaptations of the SOLAS intervention to participants and physiotherapists in order to optimise its design, uptake and delivery and (2) to determine the feasibility of trial recruitment, retention and follow-up procedures to inform the most efficient and effective study design for any future definitive trial. The secondary objectives were to explore the preliminary effects of the intervention on (3) physical function, pain, emotional and global wellbeing and (4) the process model of behaviour change compared to UP. This would inform any changes to the design of the SOLAS intervention for a future definitive trial.

A comprehensive assessment of the fidelity of intervention delivery, another key component of feasibility has been reported separately [25–27].

## Methods

### Design and setting

This was an assessor-blinded multicentre two-arm parallel cluster randomised controlled feasibility trial comparing the SOLAS intervention to UP. A cluster randomisation was chosen for practical reasons and to prevent contamination by preference of patient or PT, with each primary, community and continuing care clinic (PCCC) serving as the cluster unit. The trial was conducted in publicly funded outpatient PCCC clinics in Ireland between September 2014 and June 2016. Ethical approval was granted by University College Dublin’s Human Research Ethics Committee (LS-13-54), and the protocol was approved by the Health Service Executive (HSE) Primary Care Research Committee in March 2014 and has been published [1] and registered in Current Controlled Trials [ISRCTN49875385].

### Cluster eligibility criteria, randomisation and allocation concealment

The unit of randomisation was the PCCC clinic. PT managers in primary care areas in Dublin/North Kildare, Ireland, were approached for participation in the trial (> 1 clinic per PT manager possible) by the trial manager. Eligible clinics were identified from the clinics willing to participate. All potentially eligible clinics must have been able to provide both interventions and must have expressed willingness to send staff to the 2-day training programme required for sites randomised to the SOLAS intervention. In PCCC clinics that could not provide the SOLAS intervention on site due to a lack of suitable gym facilities, a neighbouring community site was used but all treatment was provided by PCCC clinic physiotherapists. Randomisation was performed among these eligible clinics that had confirmed willingness to participate in the study. The trial manager confirmed eligibility, willingness and ability to participate with each PT manager the day before randomisation. Eligible clinics, stratified into HSE clinics and community sites, were randomised by the statistician (RS) using R version 3.02, using restricted randomisation (permutation) to ensure balance between arms within strata. The statistician was blind to clinic identifiers during randomisation and analysis. The cluster treatment allocations were communicated by the statistician to the trial manager on the day of randomisation. The trial manager contacted each PT manager the day after randomisation after all eligibility had been checked to inform them of the allocation arm of each of their nominated sites. The PT manager then communicated the allocation arm with the relevant physiotherapy staff in each site. Patients were not approached before randomisation or the completion of PT training.

Prior to randomisation, eligible PTs in all clusters were purposively selected by PT managers to participate in the trial based on affiliation with suitable study sites, interest, experience and caseload (i.e. working full-time within chronic musculoskeletal pain service in the nominated clinic). The trial manager provided all nominated PTs with a participant information leaflet (i.e. outlined the rationale for the study and the role of consenting PTs within the trial but masked them to the feasibility trial objectives) and an opportunity to ask questions before obtaining written informed consent and completing baseline data collection of PT characteristics (including treatment expectations) prior to cluster randomisation. Due to the nature of the intervention and the pragmatic cluster trial design, it was not possible to blind PTs in either arm after randomisation.

### Participant eligibility criteria

The participant enrolment procedure developed in consultation with the clinics involved the trial manager (IJ) sending trial information and participant eligibility criteria (see Table 1) to all referring general practitioners, PTs raising awareness of the trial, screening waiting lists with the physiotherapy researchers (ET, AK) and sending potentially suitable referrals an invitation letter. Respondents were contacted by the trial manager and provided with verbal information about the study, given an opportunity to ask questions and if interested provisionally screened for eligibility over the telephone. In order to minimise selection bias during phone screening, respondents were requested not to declare their clinic details to the trial manager. Interested and potentially eligible participants were then transferred by phone to the physiotherapy researcher who sent the participant information leaflet and invitation to the local PCCC clinic. At the PCCC clinic, written informed consent for data collection was obtained prior to face-to-face screening, PT assessment and participants completing the secondary outcome measures. The participants were then informed of their treatment allocation (based on the random allocation of the PCCC clinic) by the physiotherapy researcher. The reasons for exclusion at each step in the recruitment process were recorded centrally and reviewed at weekly trial meetings between the principal investigator (DH), trial manager (IJ) and physiotherapy researchers (ET, AK) in order to minimise selection bias, particularly during face-to-face screening when treatment allocation was unblinded.

**Table 1:** Eligibility criteria for the feasibility trial

Inclusion criteria	
Diagnosis	***Osteoarthritis***
	NICE [4] working diagnosis of osteoarthritis of the hip, knee or lumbar spine defined as:
	• Age 45 years old or over, and
	• Activity related joint pain and
	• Either no morning joint-related stiffness or morning stiffness that lasts no longer than 30 min
	***Non-specific low back pain***
	Aged ≥ 30 years old with non-specific low back pain of mechanical origin with or without radiation to the lower limb
Symptom duration	Chronic (≥ 3 months)
English language	Be able to read, understand and speak English without assistance
Contact status	Access to a telephone for screening and assessment
Availability	Available to attend a 6-week start-stop group class of 1.5 h per week
***Exclusion criteria***	
Pathology	Suspected or confirmed serious spinal pathology (fracture, metastatic, inflammatory or infective diseases of the spine, cauda equina syndrome/widespread neurological disorder)
	Nerve root compromise (2 of strength, reflex or sensation affected for same nerve root)
	Lower limb arthroplasty
Past medical history	Spinal surgery or history of systemic / inflammatory disease
Current medical status	Scheduled for major surgery during treatment
Contraindications	Unstable angina / uncontrolled cardiac dysrhythmias / severe aortic stenosis / acute systemic infection accompanied by fever
Other	No confounding conditions, such as a neurological disorder, intellectual disorder or unstable psychiatric condition.
	Bladder or bowel incontinence
	People who are assessed to be at high risk of falls
	Physiotherapy in the preceding 6 months
	Unable or unwilling to attend
	Ongoing litigation related to the pain condition

### Trial interventions and physiotherapists

Treatment in both arms was provided by Chartered Physiotherapists from the participating PCCC clinics. Interventions pertain to both the cluster and individual participant level.

#### SOLAS intervention

##### Training of physiotherapists

PTs (*n* = 2 per site) randomised to the intervention arm attended 12 h standardised training over 2 days in a Dublin metropolitan university within 1 month of their scheduled start date to deliver SOLAS at their clinic. The training programme introduced PTs to the SOLAS intervention structure, content, support materials and delivery [1]. Its effectiveness in successfully supporting PTs to deliver SOLAS using a needs supportive communication style has been reported [25].

##### Intervention

Participants were required to attend a 90-min start-stop 6-week group class in the participating PCCC clinic or local community centre (if suitable gym facilities were not available) [1]. Consistent with routine PT practice and agreed during the intervention development phase [13], each group class was scheduled by PTs to run at the same time for six consecutive weeks during non-holiday periods in order to optimise participant attendance. The timing of classes was determined by PTs’ experience of providing group classes and the availability of site facilities, with each class running weekdays either late morning or early afternoon. As detailed in the trial protocol, each class comprised of 45 min education/group discussion on a specific SM topic (i.e. physical activity, pacing, healthy eating for lifestyle and balanced weight, pain management approaches including medication, pain-coping and relaxation strategies) and 45 min supervised group exercises (range of general aerobic, mobilisation and strengthening exercises for the lumbar spine, hip and knee joints) with PT guidance on exercise selection [1]. Participants were also provided with support materials to facilitate their engagement with the programme (e.g. handbook, pedometer). PTs recorded the dose of treatment provided and any harms or unintended effects experienced by any participants during the 6-week class in weekly treatment record forms developed for the trial (Additional file 1). A group class size of six participants was agreed with PTs during the SOLAS intervention development study [13]. Eleven trained PTs delivered SOLAS within the trial, with three PTs providing it on two occasions. PTs’ high fidelity to the delivery of intervention content and support materials was reported previously [27].

#### Usual individual physiotherapy

The UP treatment provided in randomised PCCC clinics was defined as individualised advice/education regarding PA, prescribed exercise, and lifestyle factors, exercise therapy and manual therapy at the PT’s discretion. They were requested not to refer participants to group-based programmes for pain management during the trial. The content and dose of treatment provided and any harms or unintended effects of treatment experienced by any participants were recorded by PTs in treatment record forms developed for the trial (Additional file 1); there was no restriction on the number of visits. Thirteen PTs delivered treatment in the UP arm.

### Outcomes and data collection

The primary feasibility outcomes related to the acceptability, demand and necessary adaptations of the SOLAS intervention and the feasibility of trial recruitment, retention and follow-up procedures to participants and PTs were evaluated using a range of qualitative and quantitative methods (Additional file 1). Participant acceptability of the SOLAS intervention compared to usual individual physiotherapy included measures of treatment expectation at baseline, attendance rates during treatment and satisfaction with treatment at follow-up (Additional file 1). The secondary outcomes were assessed using validated self-report measures of physical function, pain, emotional and global wellbeing and a range of outcomes related to the process model of behaviour change. These measures were collected at baseline/start of treatment and 2 and 6 months from baseline/start of treatment, with an additional 6-week follow-up from baseline/start of treatment included for the behaviour change outcomes. Follow-up was conducted by telephone with a blinded researcher unless participants preferred to complete them by post. Non-respondents were contacted by phone/text message on three occasions within a 3-day period to invite completion of outcomes, and if no response was obtained were posted a minimum data set outcome measure pack inviting completion at their convenience (Additional file 2).

### Sample size

The prespecified sample size consideration for the feasibility trial, as specified in the protocol [1], was to recruit 12 to 14 clusters (PCCC clinics) to test the feasibility of the intervention across a range of settings with varying staffing, facilities, equipment and clientele; aiming to recruit a minimum of six clusters in each arm, implementing two waves of recruitment, with at least six participants in each cluster per wave, for a total sample of 144 participants (72 per arm). However, a “rule-of-thumb” recommendation for pilot studies is that 30 participants per arm be enrolled to estimate parameters for future sample size calculations [28]. For the purpose of our feasibility pilot cluster trial, the sample size consideration was not based on a formal calculation, but rather on this rule of thumb designed to ensure adequately precise estimation of parameters including the standard deviation of our outcome effect sizes. As this rule of thumb was devised for simple individual-level randomised trials, we adjusted it upwards to account for a cluster design effect to prevent too much loss of precision. Assuming an intraclass correlation coefficient (ICC) of 0.03 for our secondary outcomes of physical activity, physical function and pain from a previous trial of a similar population in Ireland’s health system [29], we adjusted this rule of thumb to 36 participants per arm, and allowing for 25% loss to follow-up, we aimed to recruit 48 participants per arm (96 in total) to estimate the sample size for a definitive trial.

The specific a priori feasibility criteria to move to a definitive trial were that:

1. The SOLAS intervention was acceptable to participants and PTs and necessary adaptations were achievable
2. It was feasible to deliver the intervention with high fidelity
3. The recruitment targets of 12 clusters, a cluster size of six and a sample size of 144 participants were achieved
4. The recruitment, retention and screening procedures were successful in identifying the target population and workable for a larger trial
5. The outcome measures and follow-up procedures were acceptable to participants and operational in a larger trial
6. There was evidence of preliminary effects of the intervention on secondary outcomes and the theoretical process model of behaviour change

### Data analysis

Statistical analysis was by intention to treat according to participants’ cluster allocation. Quantitative data were coded and entered into the Statistical Package for the Social Sciences (IBM SPSS Statistics Version 24). Since this was a feasibility trial, a priori descriptive analysis of the quantitative and qualitative data were undertaken to answer the primary feasibility objectives [1]. Both participant and PT interviews were transcribed, anonymised (ET, IJ) and analysed (DMA, DH, JM) using an inductive thematic approach (see Additional file 3) [30]. Analysis of the primary feasibility objective related to trial procedures were undertaken on an interim basis after each study wave by the research team and used to inform minor protocol refinements for subsequent waves (see Additional file 1). Analysis of the secondary outcome measures was undertaken at the end of the trial and performed by the statistician who remained blinded to group identification until analysis was complete. A linear mixed model was used to examine change over time in participant outcomes between treatment groups, while adjusting for study waves and clusters. Three-level logistic or linear mixed effects models were fitted using the MIXED command, with a random intercept for each participant, and a random intercept for each cluster as a higher-level effect, using the default Newton-Raphson algorithm for restricted maximum likelihood estimation of the covariance parameters. Time and group effects, and an interaction term for time by group, were included in each model. Treatment effects were reported as model estimated marginal means with 95% confidence intervals (CI) or as medians, 1st and 3rd quartile, if skewed or a substantial floor or ceiling effect was observed. ICCs for the clusters were calculated for each endpoint. Due to the multi-joint inclusion of participants with hip, knee and lumbar spine pain, further exploratory analysis of the change over time in secondary outcomes within each of these joint pain areas was undertaken. Estimated marginal means (or medians) are also reported for each group at each time-point and mean changes from baseline to subsequent time-points are reported within groups, and between-group differences at each time-point. From logistic models, odds ratios for change from baseline to each subsequent time-point, and ratios of odds ratios to contrast the groups are reported.

## Results

### Recruitment

**Fig. 2:**
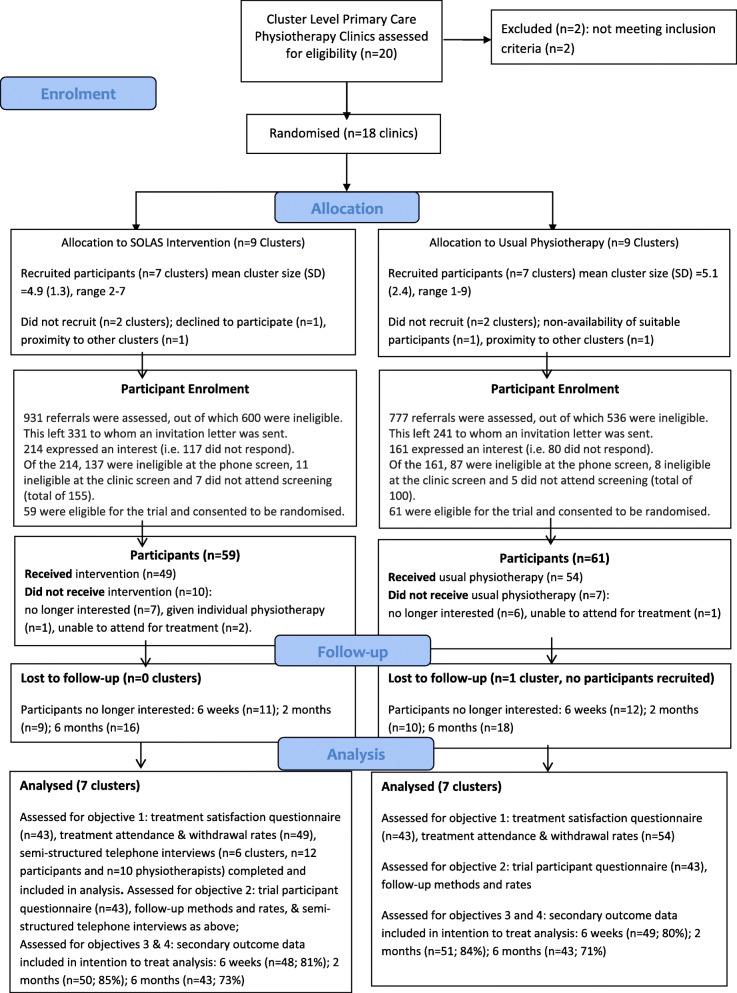
CONSORT flowchart edited for cluster and feasibility trials

Details of cluster and participant recruitment and retention are shown in Fig. 2. In January 2014, 20 clusters were invited, 18 were eligible and randomised, of which 14 randomised clinics were agreed with PT managers as available at the start of the trial and proceeded to participate in the trial (7 per trial arm). The time lag between randomisation to trial initiation for the sites within each arm was: intervention: median = 238 days, interquartile range (IQR) 232–388 days, range 232 to 391 days; UP: median = 282 days, IQR 273–373 days, range 233 to 383 days. The reasons for managers withdrawing consent for 4 clinics participation after randomisation and treatment allocation were taken for pragmatic local service reasons unforeseen prior to randomisation and not due to the outcome of randomisation as outlined in Fig. 2. Each cluster participated in two waves of recruitment (four clusters participated in pilot study) resulting in three study waves (W1–W3) between Autumn 2014 and Autumn 2015 to support the delivery of the SOLAS intervention during non-holiday periods in order to optimise attendance. In total, 120 participants (83.3%; of *n* = 144 expected) were enrolled (intervention *n* = 59; UP *n* = 61). The number of clusters and the number of participants recruited in each study wave are detailed in Table 2. Overall, the average cluster size was below the target of six participants (intervention: mean (SD) = 4.9 (1.3), range 2–7; UP: mean (SD) =5.1 (2.4), range 1–9). Three of 7 sites in the intervention arm had a cluster size of at least six participants compared to 5 of 7 sites in the UP arm. The recruitment rate in W1 was below target, which resulted in the addition of two contingency clusters for W2 and W3 and simplification of the participant invitation letter to increase its readability and clarity to potential participants, which resulted in an increase in the overall recruitment rate and mean cluster size in subsequent waves in the intervention arm.

**Table 2:** Cluster size by study wave, site and treatment arm

Wave	SOLAS intervention			Usual physiotherapy		
	Site code	Target recruitment	Cluster size recruited	Site code	Target recruitment	Cluster size recruited
**W1** Autumn 2014	A^a^	6	2	H	6	3
	B	6	6	I^a^	6	7
	C	6	4	J^a^	6	6
	D	6	5	K	6	3
				L	6	3
**Subtotal**	4	24	17	5	30	22
**Mean cluster size**			4.3			4.4
**W2** Spring 2015	B	6	4	H	6	4
	C	6	5	K	6	4
	D	6	4	L	6	7
	E^a^	6	7	M	6	6
	F	6	6	N	6	8
	G	6	5			
**Subtotal**	6	36	31	5	30	29
**Mean cluster size**			5.2			5.8
**W3** Autumn 2015	F	6	6	M	6	1
	G	6	5	N	6	9
**Subtotal**	2	12	11	2	12	10
**Mean cluster size**			5.5			5.0
**Total**	7	72	59	7	72	61

^a^Sites that participated in the pilot trial in Spring 2014 [13] participated in one recruitment wave during the feasibility trial

Between September 2014 and November 2015, 1708 referrals were identified by PTs, with 1136 (66.5%) excluded predominantly due to diagnosis (*n* = 784), age (*n* = 158), symptom duration (*n* = 53) and exclusion criteria (*n* = 133; Fig. 2). In total, 572 invitation letters were sent to potentially eligible participants, of which 375 (65.6%) responded, and 224 (59.7%) were excluded by telephone screen mainly due to preference for individual PT (*n* = 62), inability to attend SOLAS group (*n* = 30), physiotherapy in past 6 months (*n* = 22) or poor English (*n* = 31). Of the 151 invited to face-to-face screening, 31 (20.5%) were excluded (nerve root compromise *n* = 9, non-attendance *n* = 12), with 120 consenting participants recruited, representing 20.9% of invitation letters and 7% of total referrals.

### Treatment, attendance and satisfaction

The majority of participants received treatment as allocated (intervention *n* = 49; UP *n* = 54), 16 did not receive any treatment (intervention *n* = 9; UP *n* = 7) and one participant randomised to the intervention arm requested and received individual physiotherapy but remained in the intervention arm for the ITT analysis. There were no reported harms or unintended effects of treatment experienced by participants during the 6-week treatment period in either arm as recorded in the treatment record forms. The mean (SD) number of treatments received in each arm was comparable (intervention 4.3 (1.6); UP 3.8 (1.7); however, the mean (SD) duration of treatment was longer in the UP arm (7.8 (3.8) weeks) compared to the intervention arm (4.8 (1.6)). Participants in both arms reported positive ratings for overall physiotherapy care received (Table 3).

**Table 3:** Participant satisfaction and acceptability of follow-up procedures at 6-month follow-up

Participant questionnaire		SOLAS intervention (***n*** = 41)	Usual physiotherapy (***n*** = 43)
**Satisfaction**			
Over the course of treatment for your hip, knee and/or back pain how satisfied were you with your overall physiotherapy care in this study?	• Very dissatisfied	1 (3%)	2 (5%)
	• Somewhat dissatisfied	3 (7%)	2 (5%)
	• Neither satisfied nor dissatisfied	1 (3%)	2 (5%)
	• Somewhat satisfied	10 (25%)	5 (12%)
	• Very satisfied	25 (62%)	30 (73%)
Do you think the physiotherapy treatment you received in this study benefited your hip, knee and/or back pain?	• Do not know	1 (2%)	1 (2%)
	• No benefit	5 (12%)	4 (10%)
	• Some benefit	13 (33%)	16 (38%)
	• Great benefit	21 (53%)	21 (50%)
How helpful in reaching your treatment goal was the physiotherapy treatment you received in this study?	• Do not know	1 (3%)	0 (0%)
	• No benefit	3 (7%)	5 (12%)
	• Some benefit	16 (40%)	18 (43%)
	• Great benefit	20 (50%)	19 (45%)
How helpful was the advice/information you received during physiotherapy treatment in this study in helping you to manage your hip, knee and/or back pain?	• Do not know	0 (0%)	1 (2%)
	• No benefit	1 (3%)	4 (9%)
	• Some benefit	12 (29%)	12 (28%)
	• Great benefit	28 (68%)	26 (61%)
How easy/difficult has it been for you to stick to your exercise/physical activity programme since finishing treatment?	• Very difficult	6 (15%)	4 (10%)
	• Somewhat difficult	11 (27%)	11 (26%)
	• Neither difficult nor easy	5 (13%)	7 (17%)
	• Somewhat easy	13 (32%)	11 (26%)
	• Very easy	5 (13%)	9 (21%)
Would you recommend the treatment you received in this study to a relative or friend?	• Yes	39 (97%)	38 (88%)
	• No	1 (3%)	5 (12%)
Would you be happy to receive this form of treatment again?	• Yes	36 (88%)	38 (88%)
	• No	5 (12%)	5 (12%)
**Acceptability of follow-up procedures**			
How acceptable was it to you to be asked to complete the outcome measures as part of the study?	• Very unacceptable	2 (4%)	9 (0%)
	• Somewhat unacceptable	1 (2%)	2 (5%)
	• Neither acceptable nor unacceptable	3 (7%)	3 (7%)
	• Somewhat acceptable	10 (24%)	23 (55%)
	• Very acceptable	25 (61%)	14 (33%)
How much of a burden was it to you to complete the outcome measures as part of the study?	• Do not know	1 (2%)	1 (2%)
	• Great burden	2 (5%)	3 (7%)
	• Some burden	3 (7%)	9 (21%)
	• No burden	35 (86%)	30 (70%)

PT characteristics were similar between arms (see Additional file 4). The SOLAS intervention was delivered 12 times across all seven randomised clusters (five of the seven delivered it twice), in four PCCC clinics and three local community centres/gyms (see Additional file 5). Only two sites reached the target class size of 6 participants, with an overall mean class size of 4.1 (1.2) participants (min–max 2–6) showing minimal variation between waves. Eleven of 49 participants dropped out during the intervention for various reasons, but most participants (57.2%, *n* = 28) attended at least five classes corresponding to 83.3% adherence and had a treatment duration of 6 weeks (*n* = 27, 55.1%). All UP treatments were provided within all seven randomised PCCC clinics; details of treatment provided are in Additional file 6.

### Follow-up procedures

Between October 2014 and June 2016, follow-up data were obtained from 80.8% (*n* = 97) of participants at 6 weeks, 84.2% (*n* = 101) at 2 months and 71.7% (*n* = 86) at 6 months. The majority of respondents completed follow-up by phone (see Additional file 7), with the mean (SD) completion time increasing at each time-point [6 weeks: 24 (5.2) minutes (min-max 15–35); 2 months: 41 (8.9) minutes (20–60); 6 months: 44 (8.8) minutes (min-max 25–60)] as questionnaire length also increased. Most 6-month respondents found the follow-up procedures acceptable (Table 3). There was minimal missing data and no measure that participants reported difficulty completing.

### Qualitative interviews

Twelve participants who had received the intervention (8F, 4M; median (min-max) age years = 64.5, 40–79) were interviewed. Those interviewed had attended a median (IQR; min-max) of six sessions (1.8; 1–6). Ten of the 11 PTs who had delivered the intervention were interviewed. The main findings from the qualitative interviews related to the primary feasibility objectives. These ranged from the acceptability and demand of the intervention from the participant and PT perspectives, as well as the practicality and necessary adaptations to the intervention, PT training programme and trial recruitment procedures for a future definitive trial. A synopsis of these findings is presented below and supported by exemplar quotes and the number of individuals reporting each theme in Additional file 3. The qualitative studies are reported in accordance with current guidelines [31] (see Additional file 8).

#### SOLAS intervention

##### Acceptability

Participants viewed the overall experience of engaging with the intervention and resource materials very positively and had a good understanding that it was designed to educate them to take a more active role in managing their chronic musculoskeletal condition. The social aspect of the group was viewed as a key benefit by many participants. Similarly, PTs were overtly positive about their experience of providing the intervention to a mixed group, reporting it acceptable and feasible to deliver during the trial and that it addressed a need within their service and would have relevance for clients with other musculoskeletal disorders.

##### Demand

Participants reported they were likely to use some or all of the SM behaviours and related components in their daily lives; however, some participants found goal setting difficult to utilise. Key PT demands during SOLAS delivery included the volume of educational content in the first session, the perceived overemphasis on goal setting, and striking a balance in their use of language that provided appropriate direction to participants while adhering to the principle of autonomy support. Other challenges included delivering the intervention to a small group and those with inconsistent attendance or lacking motivation to engage in the exercise programme.

##### Practicality

Despite variations in facilities, gym and audio-visual equipment, PTs were satisfied that there were no practical difficulties with intervention delivery. The recruitment of enough participants was highlighted as a key issue that would need to be addressed for a future definitive trial, with the majority of PTs believing a class size of six was optimal.

##### Adaptation

Minimal changes were made during delivery, but a number of PTs made suggestions for future adaptations, particularly decreasing the educational content in session 1, potentially reducing the duration of the education component to 20–25 min, delivering the exercise component first and simplifying and adding more visuals to the handbook for those with lower literacy levels and limited time to read the materials provided.

#### Physiotherapist training

All PTs were positive about the training and feedback provided in preparation for intervention delivery, considering it acceptable in improving their ability to promote SM, while also suggesting more specific guidance and practical examples to support the demand and increase their confidence in the use of autonomy supportive language within a group setting would be beneficial in future training.

#### Trial recruitment procedures

Participants and PTs spoke very positively about their experience of trial participation. PTs expressed some concerns about the enrolment of some participants due to high levels of pain, the strict exclusion criteria and small catchment areas that limited recruitment numbers, proposing over-recruitment and the provision of some pre-group individualised treatment for a definitive trial to increase uptake.

### Secondary outcome measures and behavioural process outcomes

Participants’ baseline sociodemographic and clinical characteristics were comparable between groups (Additional file 9). There were a higher proportion of participants with a single area of pain (74.1%) than multi-joint pain (25.9%), with CLBP being the most prevalent diagnosis followed by OA knee. Both the intervention and UP arms were considered credible with similar treatment expectations.

Participant’s baseline secondary outcome (Table 4) and behavioural process outcome (Additional file 10) scores were comparable. The results of linear mixed model analysis for the continuous and categorical secondary outcomes are provided in Tables 4 and 5 respectively. Table 6 details the mean within- and between-group changes from baseline. The results of these analyses for the behavioural process outcomes are presented in Additional files 10 and 11. Further exploratory analyses of selected outcomes according to specific joint pain area are detailed in Additional file 12.

**Table 4:** Model-predicted mean (95% CI) outcomes per group over time

Outcome	Group	Time								No. clusters	Cluster ICC	Participant ICC
		Baseline		6 weeks		2 months		6 months				
**Short-form 12 physical component score (SF12-PCS)**												
Score (0–100)	Usual PT	*N* = 61	40.0 (37.8, 42.3)			*N* = 51	43.7 (41.3, 46.1)	*N* = 43	43.7 (41.2, 46.3)	7	0.0	0.56
	SOLAS	*N* = 59	39.9 (37.6, 42.2)			*N* = 50	44.1 (41.7, 46.6)	*N =* 43	42.2 (39.6, 44.8)	7		
**Pain**												
Intensity [NRS, 0–10]	Usual PT	*N* = 61	6.2 (5.5, 6.8)			*N =* 51	4.3 (3.6, 4.9)	*N* = 43	4.6 (3.9, 5.4)	7	0.0	0.38
	SOLAS	*N* = 59	5.7 (5.1, 6.4)			*N* = 50	4.8 (4.1, 5.4)	*N* = 43	4.3 (3.6, 5.1)	7		
Bothersomeness 0–10)	Usual PT	*N* = 61	3.2 (2.9, 3.5)			*N* = 51	2.6 (2.3, 2.9)	*N* = 43	2.6 (2.3, 2.9)	7	0.0	0.35
	SOLAS	*N* = 59	3.0 (2.7, 3.3)			*N* = 50	2.7 (2.4, 3.0)	*N* = 43	2.7 (2.4, 3.0)	7		
**Roland Morris disability questionnaire (RMDQ)**												
Total score (0–24)	Usual PT	*N* = 41	12.2 (10.3, 14.1)			*N* = 33	7.9 (5.9, 9.8)	*N* = 26	6.6 (4.5, 8.7)	7	0.0	0.67
	SOLAS	*N* = 34	13.0 (11,0, 15.1)			*N* = 29	9.5 (7.4, 11.7)	*N* = 25	9.4 (7.1, 11.6)	7		
**Western Ontario and McMaster Universities Arthritis Index (WOMAC) function daily living**												
Hip subscale score (0–68)	Usual PT	*N* = 14	21.5 (13.4, 29.5)			*N* = 12	23.4 (15.2, 31.6)	*N* = 10	22.4 (14.0, 30.8)	7	0.27	0.49
	SOLAS	*N* = 12	24.7 (16.3, 33.1)			*N* = 9	23.4 (14.5, 32.3)	*N* = 10	22.1 (13.4, 30.9)	7		
Knee subscale score (0–68)	Usual PT	*N* = 26	21.1 (15.7, 26.6)			*N* = 21	20.8 (15.0, 26.7)	*N* = 19	23.7 (17.7, 29.7)	7	0.13	0.50
	SOLAS	*N* = 29	24.8 (19.5, 30.2)			*N* = 27	23.9 (18.4, 29.3)	*N* = 21	26.7 (20.9, 32.6)	7		
**Hospital anxiety and depression scale (HADS)**												
Total score (0–42)	Usual PT	*N* = 61	12.5 (10.6, 14.4)			*N* = 48	10.3 (8.3, 12.3)	*N* = 43	10.1 (8.0, 12.1)	7	0.22	0.51
	SOLAS	*N* = 58	12.1 (10.2, 14.1)			*N* = 45	9.1 (7.1, 11.2)	*N* = 39	8.8 (6.7, 10.9)	7		
Anxiety scale (0–21)	Usual PT	*N* = 61	7.3 (6.2, 8.4)			*N* = 48	5.7 (4.5, 6.8)	*N* = 43	5.4 (4.2, 6.6)	7	0.05	0.66
	SOLAS	*N* = 58	7.2 (6.1, 8.4)			*N* = 45	5.2 (4.0, 6.4)	*N* = 39	5.1 (3.8, 6.3)	7		
Depression scale (0–21)	Usual PT	*N* = 61	5.1 (4.2, 6.1)			*N* = 48	4.6 (3.6, 5.6)	*N* = 43	4.57 (3.5, 5.6)	7	0.31	0.31
	SOLAS	*N* = 58	5.0 (4.0, 5.9)			*N* = 45	4.0 (2.9, 5.0)	*N* = 39	3.69 (2.6, 4.8)	7		
**Global perceived effect scale (GPE)**												
Total score (−5 to + 5)	Usual PT	*N* = 61	−2.0 (−2.8, −1.2)			*N* = 50	1.9 (1.1, 2.7)	*N* = 43	1.1 (0.2, 2.0)	7	0.0	0.0
	SOLAS	*N* = 59	−1.9 (− 2.7, 1.1)			*N* = 49	1.7 (0.9, 2.6)	*N* = 41	1.4 (0.5, 2.3)	7		
**EuroQol 5-D (EQ5D)**^**a**^												
Weighted Health Index, median [IQR], (−.59 to + 1.00)	Usual PT	*N* = 61	0.69 (0.59, 0.73)			*N* = 49	0.73 (0.66, 0.80)	*N* = 43	0.73 (0.62, 0.80)	7	N/A	N/A
	SOLAS	*N* = 59	0.69 (0.62, 0.76)			*N* = 45	0.73 (0.69, 0.76)	*N* = 39	0.69 (0.62, 0.80)	7		

^**a**^Non-normal distributions, descriptive statistics only presented

**Table 5:** Percentages for categorical outcomes

Outcome		Group	Time							
			Baseline			6 weeks		2 months		6 months
International Physical Activity Questionnaire (IPAQ)	High^a^	Usual PT	*N* = 61	32% (20, 47)	*N* = 49	37% (22, 55)	*N* = 51	36% (22, 54)	*N* = 43	30% (17, 48)
			OR from baseline:			1.26 (0.54, 2.96)		1.23 (0.53, 2.86)		0.92 (0.38, 2.26)
		SOLAS	*N* = 59	32% (19, 47)	*N* = 48	34% (20, 51)	*N* = 50	42% (27, 59)	*N* = 43	26% (14, 44)
			OR from baseline:			1.09 (0.46, 2.60)		1.29 (0.67, 3.63)		0.76 (0.30, 1.93)
		Group ratio of ORs				0.86 (0.262.92)		1.27 (0.39, 4.18)		0.83 (0.23, 3.01)
	Moderate^b^ or high	Usual PT	*N* = 61	72% (57, 83)	*N* = 49	88% (75, 95)	*N* = 51	87% (73, 94)	*N* = 43	79% (62, 89)
			OR from baseline:			2.91 (1.03, 8.21)		2.51 (0.93, 6.81)		1.44 (0.55, 3.79)
		SOLAS	*N* = 59	81% (68, 90)	*N* = 48	93% (81, 98)	*N* = 50	89% (75, 95)	*N* = 43	85% (69, 93)
			OR from baseline:			2.97 (0.85, 10.37)		1.80 (0.59, 5.44)		1.27 (0.43, 3.74)
		Group ratio of ORs				1.02 (0.20, 5.18)		0.72 (0.16, 3.18)		0.88 (0.21, 3.76)
Self-management Behaviour Questionnaire (SMBQ)	Set goals *n* (%)	Usual PT	*N* = 61	30% (18, 46)	*N* = 49	55% (37, 71)	*N* = 51	58% (40, 73)	*N* = 43	46% (29, 65)
			OR from baseline:			2.86 (1.20, 6.81)		3.23 (1.37, 7.65)		2.01 (0.81, 4.99)
		SOLAS	*N* = 59	33% (20, 49)	*N* = 48	79% (63, 90)	*N* = 50	55% (38, 72)	*N* = 43	42% (25, 61)
			OR from baseline:			8.08 (3.19, 20.45)		1.78 (1.11, 5.96)		1.51 (0.63, 3.67)
		Group ratio of ORs				2.82 (0.79, 10.08)		0.79 (0.24, 2.65)		0.75 (0.21, 2.67)
	Exercised in line with goals, *n* (%)	Usual PT	*N* = 61	22% (12, 36)	*N* = 49	52% (35, 69)	*N* = 51	55% (38, 71)	*N* = 43	49% (31, 67)
			OR from baseline:			3.93 (1.63, 9.48)		4.43 (1.85, 10.62)		3.43 (1.37, 8.59)
		SOLAS	*N* = 59	18% (10, 32)	*N* = 48	74% (57, 86)	*N* = 50	52% (35, 39)	*N* = 43	41% (25, 59)
			OR from baseline:			12.69 (4.88, 32.97)		4.81 (1.96, 11.78)		3.08 (1.21, 7.86)
		Group ratio of ORs				3.23 (0.88, 11.82)		1.09 (0.31, 3.80)		0.90 (0.24, 3.33)
	Performed small regular activity *n* (%)	Usual PT	*N* = 61	85% (72, 92)	*N* = 49	94% (83, 98)	*N* = 51	93% (81, 98)	*N* = 43	89% (74, 96)
			OR from baseline:			2.96 (0.73, 12.07)		2.27 (0.63, 8.14)		1.39 (0.41, 4.68)
		SOLAS	*N* = 59	76% (62, 86)	*N* = 48	95% (84, 98)	*N* = 50	93% (81, 97)	*N* = 43	94% (82, 98)
			OR from baseline:			5.68 (1.49, 21.61)		4.17 (1.25, 13.95)		5.15 (1.34, 19.78)
		Group ratio of ORs				1.92 (0.28, 13.35)		1.84 (0.32, 10.68)		3.70 (0.61, 22.67)
	Used mental relaxation techniques, *n* (%)	Usual PT	*N* = 61	23% (12, 38)	*N* = 49	15% (4, 30)	*N* = 51	12% (5, 26)	*N* = 43	24% (11, 43)
			OR from baseline:			0.58 (0.20, 1.67)		0.45 (0.15, 1.33)		1.05 (0.37, 2.96)
		SOLAS	*N* = 59	26% (14, 42)	*N* = 48	46% (29, 65)	*N* = 50	41% (25, 59)	*N* = 43	33% (18, 52)
			OR from baseline:			2.51 (1.02, 6.16)		1.97 (0.81, 4.78)		1.39 (0.54, 3.58)
		Group ratio of ORs				4.34 (1.08, 17.38)		4.39 (1.08, 17.84)		1.33 (0.33, 5.43)
	Did not use pain relief, *n* (%)	Usual PT	*N* = 61	7% (3, 17)	*N* = 49	24% (12, 42)	*N* = 51	31% (17, 50)	*N* = 43	38% (21, 59)
			OR from baseline:			3.98 (1.26, 12.58)		5.54 (1.79, 17.10)		7.77 (2.42, 24.95)
		SOLAS	*N* = 59	16% (8, 31)	*N* = 48	29% (15, 47)	*N* = 50	24% (12, 41)	*N* = 43	24% (12, 42)
			OR from baseline:			2.08 (0.77, 5.58)		1.60 (0.59, 4.34)		1.58 (0.57, 4.43)
		Group ratio of ORs				0.52 (0.11, 2.38)		0.29 (0.06, 1.30)		0.20 (0.04, 0.97)
	Followed healthy eating guidelines, *n* (%)	Usual PT	*N* = 61	60% (43, 76)	*N* = 49	96% (85, 99)	*N* = 51	94% (82, 98)	*N* = 43	96 (84, 99)
			OR from baseline:			15.26 (3.75, 62.18)		9.87 (2.79, 34.88)		14.02 (3.27, 60.18)
		SOLAS	*N* = 59	80% (62, 91)	*N* = 48	100% (0,100)	*N* = 50	97% (88, 99)	*N* = 43	100% (0, 100)
			OR from baseline:			N/A		8.13 (1.91, 34.54)		N/A
		Group difference				N/A		0.82 (0.13, 5.12)		N/A

*IPAQ Categorical Score* ^a^High*:* participants achieving either (a) vigorous-intensity activity on at least 3 days achieving a minimum total physical activity of at least 1500 MET-minutes/week *or* (b) 7 or more days of any combination of walking, moderate-intensity or vigorous-intensity activities achieving a minimum Total physical activity of at least 3000 MET-minutes/week. ^b^Moderate: The pattern of activity to be classified as “moderate” is either of the following criteria: (a) 3 or more days of vigorous-intensity activity of at least 20 min per day *or* (b) 5 or more days of moderate-intensity activity and/or walking of at least 30 min per day *or* (c) 5 or more days of any combination of walking, moderate-intensity or vigorous-intensity activities achieving a minimum total physical activity of at least 600 MET-minutes/week

**Table 6:** Mean (95% CI) within- and between-group changes for secondary outcomes

Outcome	Group	Time		
		6 weeks	2 months	6 months
**SF12-PCS**				
Score	Usual PT		3.68 (1.39, 5.97)	3.71 (1.27, 6.14)
	SOLAS		4.18 (1.86, 6.49)	2.26 (−0.18, 4.71)
	Group difference:		−0.49 (−3.75, 2.76)	1.44 (−2.01, 4.89)
**Pain**				
Intensity (NRS)	Usual PT		−1.86 (−2.60, −1.13)	−1.50 (−2.28, −0.72)
	SOLAS		−0.96 (−1.71, −0.22)	−1.39 (−2.17, −0.60)
	Group difference:		−0.90 (−1.94, 0.15)	−0.11 (−1.22, 0.99)
Bothersomeness	Usual PT		−0.65 (−0.98, −0.31)	−0.61 (−0.96, −0.25)
	SOLAS		−0.31 (−0.65, 0.02))	−0.29 (−0.65, 0.06
	Group difference:		−0.33 (−0.81, 0.14))	−0.31 (−0.81, 0.19
**RMDQ**				
Total score	Usual PT		−4.34 (−6.02, − 2.66)	−5.59 (−7.43, −3.76)
	SOLAS		−3.46 (−5.26, − 1.66)	−3.65 (−5.55, − 1.75)
	Group difference:		−0.88 (−3.35, 1.58)	− 1.94 (−4.59, 0.70)
**WOMAC function daily living**				
Hip subscale score	Usual PT		1.91 (−3.61, 7.43)	0.88 (−5.03, 6.79)
	SOLAS		−1.30 (−7.56, 4.96)	−2.58 (−8.59, 3.44)
	Group difference:		3.21 (−5.13, 11.55)	3.45 (−4.98, 11.89)
Knee subscale score	Usual PT		−0.32 (−5.33, 4.69)	2.54 (−2.66, 7.73)
	SOLAS		−0.97 (− 5.44, 3.49)	1.91 (−2.99, 6.81)
	Group difference:		0.65 (−6.06, 7.37)	0.63 (−6.51, 7.77)
**HADS**				
Total score	Usual PT		−2.20 (−3.66, −0.70)	− 2.41 (− 3.95, − 0.87)
	SOLAS		− 3.00 (−4.53, −1.47)	−3.36 (−4.97, − 1.74)
	Group difference:		0.82 (−1.30, 2.95)	0.95 (−1.29, 3.18)
Anxiety scale	Usual PT		−1.67 (− 2.57, − 0.77)	−1.91 (− 2.85, − 0.97)
	SOLAS		−2.03 (− 2.96, − 1.10)	−2.10 (− 3.09, − 1.12)
	Group difference:		0.36 (− 0.93, 1.66)	0.19 (− 1.16, 1.55)
Depression scale	Usual PT		− 0.55 (− 1.40, 0.30)	−0.56 (− 1.44, 0.32)
	SOLAS		−0.99 (− 1.87, − 0.12)	− 1.28 (− 2.21, − 0.36)
	Group difference:		0.44 (− 0.78, 1.66)	0.72 (− 0.55, 2.00)
**GPE**				
Total score	Usual PT		3.85 (2.98, 4.72)	3.05 (2.14, 3.95)
	SOLAS		3.65 (2.77, 4.52)	3.29 (2.36, 4.21)
	Group difference:		0.21 (−1.03, 1.44)	−0.24 (− 1.53, 1.06)
**EQ5D**				
Weighted Health Index	Usual PT		+ 0.08	+ 0.06
	SOLAS		+ 0.01	+ 0.04
	Group difference:		0.06	0.02

### Changes in secondary outcomes

There were improvements in the mean scores for most secondary outcomes at 2 and 6 months for the overall sample and within each diagnostic subgroup, apart from the WOMAC scores which showed minimal change for both OA hip and knee participants. There were small between-group mean differences, apart from the NRS-pain intensity at 2 months and RMDQ at 6 months which approached their MCID values in favour of the UP group. However, the proportion of responders [≥30% drop from baseline] at 2 months was comparable in both groups for the RMDQ [UP 57.6%; SOLAS 58.7%] and NRS-pain intensity scale [UP: 47.1%; SOLAS: 44%].

There was an increase from baseline in both groups in the proportion of participants engaging in moderate or high levels of PA and SM behaviours related to physical activity at all time-points with the group ratio of ORs favouring SOLAS at all time-points. There was an increase in the proportion of participants using mental relaxation techniques only in the SOLAS group with large group ratios of ORs at 6 weeks (4.34) and 2 months (4.39), while the non-use of pain relief increased at 6 weeks in both groups and continued to rise in the UP group only, the group ratio of ORs were small at all time-points. Finally, there were large increases in the proportion of participants who reported eating healthily at all time-points in both groups.

### Changes in the process model of behavioural change

At 6 weeks, there were improvements in the SDT-based determinants of SM behaviour with the between-group mean difference in change from baseline in favour of SOLAS for the measures of perceived competence [PCQ-PA mean, 95% CI = − 0.37, − 0.99, 0.25; PCQ-SM = − 0.46, − 1.07, 0.16], and motivation to participate in physical activity [BREQ-RAI = − 0.71, − 1.78, 0.36] and to self-manage [TSRQ-RAI = − 1.19, − 2.96 to 0.59]. There were also small changes at 6 weeks in favour of SOLAS for pain catastrophising [PCS = − 1.02; − 2.96, 5.00], but in favour of UP for fear (TSK = − 0.71, − 1.99, 0.56). At 2 and 6 months, the intervention effects on perceived competence and autonomous motivation gradually reduced, while the effects on controlled and amotivation remained stable or increased with small between-group differences in favour of SOLAS at 6 months. Changes in pain catastrophising and fear increased in both groups over time, with small between-group mean differences evident (Additional file 11).

## Discussion

This is the first feasibility trial of a group-based theoretically informed complex self-management intervention for both OA and chronic LBP that has evaluated its acceptability alongside testing the proposed trial procedures from the perspectives of both healthcare providers and patients. Preliminary effects of the intervention were also explored, as was the proposed process model of behaviour change.

### Feasibility: acceptability, demand and necessary adaptations of the SOLAS intervention

The findings of the qualitative interviews and self-report measures demonstrated that the SOLAS intervention content, support materials and group-based mode of delivery were acceptable and appropriate to participants with OA and CLBP and physiotherapists alike. These findings are reinforced by our previous report of high fidelity to these elements of the intervention [27]. Feasible adaptations for a future definitive trial include simplifying the education content of the first session to increase its acceptability and fidelity and ensuring the materials are suitable for participants with low health literacy.

Fifty seven percent of participants attended five out of six SOLAS classes. This is a higher attendance rate than other RCTs of the 6-week group interventions for CLBP delivered in the Irish health service [32, 33], and comparable to the ESCAPE-knee pain intervention in the UK health service [15]. However, the small class sizes with an average of four participants rather than our target of six and inconsistent participant attendance placed demands on the practicality of PTs’ delivery of the intervention with high fidelity [27], and thus challenge the viability of a future definitive trial of this intervention and its future implementation within the health system, as discussed below.

### Feasibility: trial recruitment, retention and follow-up procedures

The trial was successful in recruiting 14 clusters demonstrating the strong partnership between the research team and PCCC areas established during the development phase [13]. Nonetheless, four clusters withdrew consent after randomisation and treatment allocation for reasons unforeseen at the outset of the trial (i.e. proximity to other clusters and non-availability of suitable participants), and which are partly due to the time lag of at least 9 months between randomisation and commencement of the trial in some of these sites. Overall, 21% of potential participants sent invitation letters were recruited, which is within the range of other trials of group-based programmes for these populations [15, 32–35]. Furthermore, the recruitment protocol successfully enrolled participants with OA of the hip, knee, lumbar spine and CLBP, with the latter being the most prevalent in line with population data. In contrast to the FASA intervention, which restricted recruitment to individuals with OA aged at least 50 years [12], our findings have demonstrated the feasibility of enrolling and retaining younger participants with CLBP to a group-based programme alongside older people with OA. Nonetheless, the average cluster size of five participants in both arms, the average class size of four participants and the overall recruitment rate were below the target of 144 required to demonstrate feasibility, but enough for a sample size calculation for a definitive trial as discussed below.

Despite the recruitment protocol being embedded within the health system and developed in partnership with PTs [13], several challenges beyond our control impacted upon its success. First, the research ethics committee requirement that potentially suitable participants contact study staff resulted in a 66% response to the invitation letter. Second, the need for individual participant consent for data collection prior to enrolment rather than employing a more pragmatic service-based quality improvement protocol that would have automatically enrolled all patients at the cluster level negatively impacted on our ability to reach our target population. Third, participant recruitment was outside routine PT practice and was conducted by non-clinical research PTs, thus potentially increasing the complexity for patients in accessing physiotherapy.

Despite increases in the recruitment rate, cluster size and response to invitation letters across waves as study procedures were improved, further protocol changes would be required to ensure recruitment targets and the optimal class size of six are achievable in any future definitive trial. For example, the time-consuming paper-based exclusion of most referrals for physiotherapy due to diagnosis and age is not an effective use of trial resources or feasible for a definitive trial. Recruitment efficiency would be increased if computer-generated identification codes were available in Ireland’s health service as in other jurisdictions. Furthermore, despite high levels of reported participant satisfaction and acceptability of the SOLAS intervention, some of the main reasons for excluding participants at the phone screen stage were their preference for individual PT, their inability to commit to the 6-week class and their poor English fluency, which challenge the feasibility of a future definitive trial within Ireland’s public health system. The high percentage of potentially suitable participants expressing a preference for individual treatment is an accurate reflection of the real-world setting in which SOLAS would be offered to interested patients in future clinical practice and illustrates that a definitive trial based on individual patient randomisation would not have a higher likelihood of successful recruitment than the cluster design utilised in this feasibility trial. Further pragmatic obstacles to individual participant randomisation in a definitive trial are the inconsistency in the availability of sufficient physiotherapists within these relatively small PCCC clinics (1–3 musculoskeletal PTs per clinic) who could provide both treatment arms independently, and the need to train 2 PTs per site in the SOLAS arm to mitigate against frequent maternity, parental, sick and study leave who are not replaced if staff are absent.

Since the completion of this trial, the importance of patient and public involvement (PPI) in research has become increasingly recognised in Ireland [36, 37]. Therefore, the development of a revised recruitment pathway for a definitive trial would warrant further PPI engagement to address barriers and optimise enablers to participation in the group-based class arm in particular [38–40].

The response rate at 2 months was acceptable, but the 6-month response rate at 72% was below the assumed loss to follow-up rate of 25%. It is likely that despite the support of our researcher and the preference of the majority of respondents for telephone follow-up, the average 41 min to complete it at 2 months and the addition of the CSRI at 6 months were off putting to some non-respondents. Therefore, the number of follow-up points and multiple outcome measures that accounted for each joint condition and the complex behaviour change process would need to be reduced to maximise response rates and optimise follow-up procedures for a future definitive trial as discussed below. Furthermore, an additional strategy to protect against loss to follow-up would be to offer a telephone interview to collect data points required for analysis of the primary outcome only, in the case of participant non-response.

### Feasibility: design of a definitive trial

The above findings inform the most efficient and effective study design for any future definitive trial. Some expert trialists have raised problems with the cluster trial design due to compromised concealment of allocation and improper randomisation (i.e. clusters are recruited and randomised and then participants are enrolled as occurred in this feasibility trial causing differential recruitment and imbalance between groups) [41], arguing in favour of individual participant randomisation [42]. There is no evidence that the SOLAS feasibility trial displayed selection bias as the recruitment rate and cluster size were comparable between arms across the three study waves, the two arms were balanced at baseline for all sociodemographic variables for both participants and physiotherapists and there was minimal crossover from the intervention to the control arms during the treatment phase (i.e. *n* = 1 participant), indicating minimal contamination. Nonetheless, the authors have considered the feasibility of incorporating recommendations to cluster trial recruitment processes to minimise these challenges in the design of a future definitive trial [41]. These include the screening and enrolment of all participants prior to cluster randomisation or (if this is not possible) the randomisation of clusters after the enrolment of the first participant. While ideal, these solutions would be challenging in Ireland’s health system due to the nature of the SOLAS intervention (i.e. the impossibility of blinding PTs and the 2-month lead in time for PT training that would cause lengthy waiting times for participants after cluster randomisation), and the complexity of the multi-stakeholder recruitment process described previously. A further recommendation to use blinded independent recruiters who are separate from the allocation process [41] could be used in a definitive trial with greater resources than our feasibility trial where staff involved in the central recruitment processes were blinded to the cluster allocation of individual participants and followed the same protocol regardless of cluster allocation.

In determining the proposed sample size for a definitive trial based on our secondary outcome results, the authors selected the SF12-PCS as the primary outcome with a primary endpoint of 6 months based on other RCTs of self-management for chronic musculoskeletal pain and other chronic conditions [43, 44]. The minimal clinical important change for the SF12-PCS is reported as 3.2 [45], giving an estimated sample size for a definitive trial using a cluster design with a cluster size of six, using the observed cluster ICC (conservatively, 0.01) and estimated standard deviation for baseline-adjusted 6-month SF12-PCS (8.49), a definitive trial would require 117 participants per arm to achieve 80% power at a type I error rate of 0.05. A further increase of at least 25% of this sample size target would be required for retention at 6 months as discussed above resulting in a recruitment target of 156 participants per arm. Based on the previously discussed challenges to the recruitment, retention and follow-up of participants in this feasibility trial at both the cluster and total sample size levels, the likelihood of reaching this recruitment target in a definitive trial in Ireland’s health system is low.

### Changes in secondary outcomes

The finding of comparable small effects for both SOLAS and individual PT for the majority of secondary outcomes is consistent with our rapid review [9] and other systematic reviews of education and exercise SM programmes for OA [7] and LBP [8]. The larger improvements in pain intensity in the UP group at 2 months and in LBP-related functional disability at 6 months could be associated with the multi-modal treatments utilised targeting analgesia, including clinical guideline endorsed manual therapy [5]. A recent trial of SDT-driven individual physiotherapy for LBP found limited effects for pain, function or quality of life compared to usual PT, with similar group differences to the current study [29]. The minimal change in the WOMAC-physical function subscale for OA hip or knee participants may reflect its poor responsiveness compared to other measures or physical performance tests and warrants omission in any future definitive trial [46], with the use of only the SF-12 for all diagnostic subgroups to further reduce respondent burden.

The SOLAS intervention maximal dose of 9 h over 6 weeks, which was agreed with PTs during the development phase and found to be acceptable in this feasibility trial, is relatively low compared to other group-based interventions (including FASA) that have shown larger between and within group effects on pain, function and quality of life outcomes for OA knee [15, 47, 48]. Conversely, clinical LBP guidelines recommend group-based exercise programmes that promote self-management but were unable to recommend the intensity of the programme [5].

### SOLAS process model of behaviour change

The effect of the intervention on LBP-related determinants was minimal, with weak effects in the full sample for pain catastrophising and no effect on fear at completion of the 6-week programme [5, 49]. The measurement of fear avoidance may have been underestimated due to the use of the 6-item activity avoidance subscale of the TSK-11 [50] to reduce respondent burden. Nonetheless, it is proposed that these variables should be removed from the process map of behaviour change given their tentative evidence, its complexity, and the multi-joint focus of the intervention.

In line with the assumptions of SDT, there were small changes in participants’ perceived competence and motivation for both PA and self-management that favoured SOLAS at week 6, but these changes alone were not enough to promote long-term increases in participant behaviour. These findings are consistent with previous literature and suggest sustained increases in autonomous motivation may be required for behaviour change [51, 52]. Although PTs underwent training and were deemed competent to deliver SOLAS within the feasibility trial, they struggled to effectively utilise specific strategies related to goal setting [25, 26]. This is noteworthy as a collaborative goal-setting process between a health care professional and patient is likely to be important in increasing and sustaining a patient’s autonomous motivation and competence for the particular behaviour [53–55]. Additionally, some PTs in the qualitative interviews felt they needed further training to augment their use of autonomy supportive language (i.e. flexible and suggestive rather than pressurising language) when delivering SOLAS, an important communication technique for promoting autonomous motivation [56]. This requirement was reinforced by independent observers who rated PTs’ average use of autonomy supportive language as moderate (4.2 on a 7-point Likert scale) [25].

The small increases found in subjectively measured PA and in the SM behaviours related to PA within SOLAS up to 2 months provide preliminary evidence of its effect on these behaviours. PTs’ overall moderate fidelity to the intervention BCTs and their inability to deliver all 26 BCTs targeting PA within the trial may have contributed to these small effects [26]. If a definitive trial is to take place, first, the core intervention BCTs must be identified and second, training enhancements are required to target PTs’ use of particular BCTs.

The limitation of self-report measures of PA is well recognised in the literature, due to recall bias, social desirability bias and poor correlation with objective measures [57]. There is currently no evidence that an increase in self-reported PA is associated with improvements in pain and disability outcomes for OA [57] and LBP [58], but the quality of current research is low, the majority of current interventions lack a strong theoretical basis and have failed to evaluate treatment fidelity and the findings of the current study shed some light on these elements for future interventions targeting PA.

The higher use of mental relaxation techniques in the SOLAS group at 2 and 6 months may reflect the greater focus on the uptake of these skills within the intervention and may be associated with the consistent small reductions in HADS subscale scores in favour of SOLAS, suggesting the relatively short time focusing on this SM skill could be increased given the moderate levels of anxiety and depression of the sample at baseline. Conversely, the marginally lower use of pain relief techniques in the UP group at 2 and 6 months could be related to the greater reduction in pain intensity at 2 months in this group.

The major strengths of this feasibility trial relate to the use of a comprehensive range of quantitative and qualitative methods and the inclusion of a high number of clusters across a range of sites and geographical areas to address clearly defined feasibility objectives and a priori criteria for moving to a definitive trial from both participant and PT perspectives. The design of the feasibility trial was guided by the MRC framework, underpinned by behaviour change theory and extensive stakeholder engagement, and its reporting conforms to CONSORT guidelines for feasibility [24] and cluster trials [59] (see Additional file 13). There were also some limitations that should be acknowledged including the unforeseen withdrawal of consent of four clusters after treatment allocation, below target recruitment rate and the high number of secondary outcomes that probably contributed to the below expected response rate at 6 months. While adherence to the intervention SM skills (apart from specific exercise) was measured by an unvalidated researcher-designed questionnaire, consistent with many similar studies [60], the qualitative participant interviews of participants enactment of SM skills supported these findings and could contribute to its future validation. A self-report measure was used to assess participant PA, and the inclusion of a user-friendly low-cost objective measure of PA in any future definitive trial is warranted. It was not possible to blind participants or PTs due to the nature of the study, and we did not interview participants who did not complete the 6-month follow-up or those with low attendance rates.

## Conclusions

The findings have demonstrated that the complex, group-based, theory-driven SOLAS intervention is acceptable to PTs and patients with OA and CLBP and has preliminary evidence of small effects on the secondary outcomes and the process map of behaviour change comparable to individual physiotherapy in its current format and dose. Minor changes to the intervention content, underpinning process model, BCTs and PT training programme have been identified to optimise its design, uptake and delivery for evaluation in a definitive trial. However, the likelihood of recruiting enough participants for a definitive cluster trial in Ireland’s current primary care service is low given the significant constraints on participant identification, recruitment and enrolment procedures identified in this study thus rendering a definitive trial unfeasible.

## Supplementary information

**Additional file 1.** Primary Feasibility Outcomes and Measures.

**Additional file 2.** Secondary Outcomes and Process Model of Behaviour Change Measures.

**Additional file 3.** Qualitative Interview Methods and Results.

**Additional file 4.** Physiotherapist Baseline Characteristics.

**Additional file 5.** SOLAS Intervention Sites, Physiotherapists and Participants.

**Additional file 6.** Usual Physiotherapy Treatment.

**Additional file 7.** Methods of Follow-up.

**Additional file 8.** Consolidated Criteria for Reporting Qualitative Research (COREQ) Guidelines for Physiotherapist and Participant interviews.

**Additional file 9.** Baseline sociodemographic variables.

**Additional file 10.** Model-predicted mean (95% CI) behaviour change process model outcomes per group over time.

**Additional file 11.** Mean (95% CI) within and between group changes for behaviour change process outcomes.

**Additional file 12.** Exploratory Analysis by Joint Pain Condition.

**Additional file 13.** CONSORT Checklist.

## Data Availability

All data generated and analysed during this study are included in the published article and its supplementary information files.

## Abbreviations

BCT: Behaviour change technique
BREQ: Behaviour Regulation Exercise Questionnaire
CI: Confidence interval
CSRI: Client Services Receipt Inventory
CLBP: Chronic low back pain
EQ5D: EuroQol 5-D Weighted Health Index
FASA: Facilitating Activity and Self-management in Arthritis
GPE: Global perceived effect scale
HADS: Hospital Anxiety and Depression Scale
ICC: Intraclass correlation coefficient
IPAQ: International Physical Activity Questionnaire
MRC: Medical Research Council
NRS: Numeric Rating Scale
OA: Osteoarthritis
PA: Physical activity
PCCC: Primary, community and continuing care
PCQ-PA: Perceived Competence Questionnaire for physical activity
PCQ-SM: Perceived Competence Questionnaire for self-management
PCS: Pain Catastrophising Scale
PPI: Patient and public involvement
PT: Physiotherapy/physiotherapist
RMDQ: Roland Morris Disability Questionnaire
SDT: Self-determination theory
SF12-PCS: Short Form-12 Physical Component Score
SM: Self-management
SOLAS: Self-management of osteoarthritis and low back pain through activity and skills
SMBQ: Self-management Behaviour Questionnaire
TSRQ: Treatment Self-Regulation Questionnaire
TSK-11: Tampa Scale of Kinesiophobia Activity Avoidance Subscale
UP: Usual individual physiotherapy
W: Wave
WOMAC: Western Ontario and McMaster Universities Arthritis Index
