# Supplementary material for: Feasibility cluster randomised controlled trial evaluating a theory-driven group-based complex intervention versus usual physiotherapy to support self-management of osteoarthritis and low back pain (SOLAS)
Source: Trials. 2020 Sep 23;21:807. doi: 10.1186/s13063-020-04671-x (PMC7510107; doi:10.1186/s13063-020-04671-x)
Supplement: Supplementary file 1 — Additional file 1. Primary Feasibility Outcomes and Measures. [file 13063_2020_4671_MOESM1_ESM.docx]

**Additional file 1:** **Primary Feasibility Outcomes: Acceptability, Demand and Necessary Adaptations of the SOLAS Intervention and the Acceptability and Feasibility of Trial Recruitment, Retention and Follow-up Procedures**

| **Variable** | **Measure and items** | **Details** | **Measurement point, trial arms** |
| --- | --- | --- | --- |
| Expectation of treatment  [Participants and PTs] | Expectation of treatment scale  4-items  10 point numeric rating scales ranging from ‘not at all helpful’ to ‘extremely helpful’. | Participants and PTs rate how helpful they believe both the SOLAS intervention and usual individual PT treatment will be for people with OA and CLBP. | Baseline only |
| Attendance rate at weekly SOLAS intervention class or usual individual physiotherapy  [Participants] | Treatment record forms  developed for this trial [27] | PTs completed weekly treatment record forms which captured participant attendance, rates, reasons for non-attendance, early withdrawal or any harms or unintended effects of treatment | During delivery of SOLAS intervention or usual individual physiotherapy |
| Satisfaction with outcome and care  [Participants] | Satisfaction questionnaire*  2-items  Measured using 5-point numeric rating scales. | Participants rate their satisfaction with PT care received in the trial. | 2 months,  6 months |
| Acceptability, demand and necessary adaptations of the SOLAS intervention and trial procedures  [Participants] | Purposive sample of participants who received the SOLAS intervention invited to participate in individual qualitative semi-structured telephone interviews with Physiotherapy Researcher | Participants discussed their experience of the SOLAS intervention and trial participation and necessary adaptations to optimise acceptability and uptake | Post completion of 6 month follow-up |
| Acceptability, demand and necessary adaptations of the SOLAS intervention and trial procedures  [PTs] | All PTs who delivered the SOLAS intervention invited to participate in individual qualitative semi-structured telephone interviews with Qualitative Researcher | PTs’ discussed their experience of delivering the SOLAS intervention, PT training programme and trial recruitment procedures and necessary adaptations to optimise acceptability and uptake | Within two weeks of completion of SOLAS intervention delivery |
| Acceptability of trial recruitment and follow-up procedures  [Participants] | Brief questionnaire developed for this trial*  11-items  Measured using 5-point numeric rating scales and yes/no responses. | Participants rate their acceptability and perceived burden of trial participation and outcome measure completion. | 6 months |
| Feasibility of recruitment procedures | Cluster recruitment | Calculation of recruitment rate and comparison to trial protocol: minimum of 6 clusters per arm participating in two waves of recruitment | After each study wave |
|  | Participant recruitment | Calculation of participant recruitment per study arm, per cluster and overall and comparison to trial protocol: 6 participants in each cluster per wave [i.e. 144 participants, 72 per arm] | After each study wave |
|  | Reasons for refusal | Assess number and reasons for refusal and if they can be addressed for definitive trial | After each study wave |
|  | Eligibility criteria suitability | Assess number and reasons for exclusion at each stage in the recruitment process and if any changes needed to eligibility criteria for definitive trial | After each study wave |
|  | Success and practicality of screening process | Trial protocol specified three stage screening of waiting list, telephone and face-to-face screening involving PTs and Researchers. Assess if any changes needed for definitive trial | After each study wave |
| Feasibility of participant retention procedures | Reasons for withdrawal | Trial protocol specified retention of 36 participants per arm. Assess number and reasons for withdrawal | After each study wave |
| Feasibility of follow-up procedures | Response rate    Methods of follow-up  Time to complete telephone follow-up  Level of missing data | Trial protocol specified 25% loss of follow-up overall  Proportion of participants completing phone, post or email follow-up  Researcher time to administer outcome measure completion recorded at each follow-up point  Missing data for all measures recorded by the Researcher | After each study wave |
