# Supplementary material for: Feasibility cluster randomised controlled trial evaluating a theory-driven group-based complex intervention versus usual physiotherapy to support self-management of osteoarthritis and low back pain (SOLAS)
Source: Trials. 2020 Sep 23;21:807. doi: 10.1186/s13063-020-04671-x (PMC7510107; doi:10.1186/s13063-020-04671-x)
Supplement: Supplementary file 2 — Additional file 2. Secondary Outcomes and Process Model of Behaviour Change Measures. [file 13063_2020_4671_MOESM2_ESM.docx]

**Additional file 2 Secondary Outcomes**

| **Variable** | **Measure and items** | **Details** | **Reliability where available** | **Administration point, trial arms** |
| --- | --- | --- | --- | --- |
| **Secondary Outcomes** | | | | |
| Physical functioning | Short Form-12 Physical Component Score ^[^[^61^](#_ENREF_8)^]^ [SF-12 PCS]*  12-items | Patients rate health and the limitations caused by physical problems on 5 point Likert scales ranging from all of the time to none of the time. | Internal consistency: 0.77^[62]^ | Baseline,  2 months,  6 months |
| Low back pain-specific functional disability | Roland-Morris Disability Questionnaire  ^[63^] RMDQ]*  24-items | Patients respond to statements which match their experience of low back pain on the day of completion on a dichotomous scale. | Internal consistency: 0.91^[63]^ | Baseline,  2 months,  6 months |
| Osteoarthritis specific-functional disability | WOMAC Function Daily Living Hip and/or Knee Subscale ^[^[^6^](#_ENREF_5)^4]*^  17-items | Patients rate difficulty with hip and/or knee in performing various everyday activities. | Internal consistency for the subscales range: 0.83-0.96^[^[^65^](#_ENREF_6)^]^ | Baseline,  2 months,  6 months |
| Pain intensity | Numeric Rating Scale NRS] ^[66]^*  1-item | Patients rate their level of pain in the past week on an 11-point numeric scale. | Inter-rater reliability: 0.56^[67]^ | Baseline,  2 months,  6 months |
| Pain bothersomeness | Single item on a numeric rating scale ^[^[^68^](#_ENREF_1)^]*^  1-item | Patients rate how bothered they’ve been by their pain in the last week on a 5-point scale ranging from not at all to extremely. | Internal consistency: 0.79^[68]^ | Baseline,  2 months,  6 months |
| Mood/  Emotional functioning | Hospital Anxiety and Depression Scale [HADS] ^[69]^  14-items | Patients rate agreement with items related to anxiety or depression, such as “I feel tense or ‘wound up’” on a 4-point scale. | Internal consistency for the subscales range: 0.78-0.86^[^[^7^](#_ENREF_16)^0]^ | Baseline,  2 months,  6 months |
| Quality of life  [Health state] | EuroQol 5-D Weighted Health Index^[^ [EQ-5D] ^[71]^  5-items | Patients describe their current health state under five domains: mobility, self-care, usual activities, pain/discomfort, anxiety/depression. | Test-retest reliability range for the subscales range: 0.70-0.85^[72]^ | Baseline,  2 months,  6 months |
| Global impression of change | Global Perceived Effect Scale [GPE]*^[ 73]^  1-item | Patients rate on an 11-point scale the level of change (positive or negative) in their hip, knee and/or back pain between now and when the pain episode started ranging from vastly worse to vastly recovered. | Test-retest reliability excellent: intraclass correlation coefficient: 0.90-0.99^[74]^ | 2 months,  6 months |
| **Process of behaviour change outcomes** | | | | |
| Perceived competence to engage in physical activity | Perceived Competence Questionnaire for physical activity [PCQ-PA^[75]^  4-items | Patients rate their perceived ability to exercise and/or engage in physical activity regularly and in the long term on a 7-point scale ranging from not at all true to very true | Internal consistency range: 0.80-0.94^[75]^ | Baseline,  Week 6,  2 months  6 months |
| Perceived competence for self-management | Perceived Competence Questionnaire for self-management [PCQ-SM] ^[75]^  4-items | Patients rate their perceived ability to manage their pain regularly and in the long term on a 7-point scale ranging from not at all true to very true | Internal consistency range: 0.80-0.94^[^[^75^](#_ENREF_71)^]^ | Baseline,  Week 6,  2 months,  6 months |
| Autonomous, controlled and amotivation  to participate in physical exercise | Behaviour Regulation Exercise Questionnaire  [BREQ] ^[^[^76^](#_ENREF_68)^]^  10-items | Patients answer items related to their motivation to engage in physical exercise on a 5-point scale ranging from not true for me to very true for me. | Internal consistency: 0.89^[77]^ | Baseline,  Week 6,  2 months,  6 months |
| Autonomous, controlled and amotivation  to follow PTs advice to self-manage) | Treatment Self-Regulation Questionnaire  [TSRQ] ^[^[^7^](#_ENREF_66)^8]^  9-items | Patients rate their agreement with statements on why they follow their PT’s advice on a 7-point scale ranging from not true for me to very true for me. | Internal consistency: 0.76^[^[^78^](#_ENREF_67)^]^ | Baseline,  Week 6,  2 months,  6 months |
| Pain catastrophizing | Pain catastrophizing scale [PCS] ^[79]^  13-items | Patients indicate their agreement with items following the question ‘when in pain’ such as I anxiously want the pain to go away’ on 5-point scales ranging from ‘not at all’ to ‘all of the time’. | Internal consistency: 0.95^[80]^ | Baseline,  Week 6,  2 months,  6 months |
| Fear | Tampa Scale of Kinesiophobia Activity Avoidance Subscale [TSK-11] ^[81]^  6-items | Patient rate beliefs about their pain on a 4-point scale ranging from strongly disagree to strongly agree. | Internal consistency: 0.91^[82]^ | Baseline,  Week 6,  2 months,  6 months |
| **Target Behaviours** | | | | |
| Physical activity | International Physical Activity Questionnaire [IPAQ]* ^[83]^  7-items | Patients provide time spent undertaking vigorous/moderate physical activity, walking and being sedentary in the last 7 days. | Test-retest reliability range: 0.46-0.96**^[83]^** | Baseline,  Week 6,  2 months,  6 months |
| SOLAS self-management strategies | Self-management Behaviour Questionnaire [SMBQ]*  6-items | Patients describe adherence to target self-management behaviours in the past week on yes/no and number of day scales. | No reliability data available | Baseline,  Week 6,  2 months,  6 months |
| **Costs** | | | | |
| Resource utilisation costs of the participant | Client Services Receipt Inventory [CSRI] ^[84]^  20-items | Patients recall costs related to their condition and its treatment: healthcare, labour productivity changes, costs borne by patients and their families and state benefits received. | Concordance correlation: 0.756^[85]^ | Baseline,  6 months |

Baseline face-to-face interview with a Physiotherapy Researcher; week 6, 2 months and 6 months telephone interview with a blinded Researcher. The time for completion by telephone was documented. Participants had the option to complete outcome assessment by post if preferred.

*Minimum Data Set: Non-respondents were contacted by phone/text message on three occasions within a three day period; if no response was obtained the minimum data set was posted with a pre-paid envelope.

**References**

61. Ware JE Jr, Kosinski M, Keller SD. A 12-item short-form health survey: construction of scales and preliminary tests of reliability and validity. Med Care. 1996. https://doi.org/10.2307/3766749.

62. Luo X, George ML, Kakouras I, Edwards CL, Pietrobon R, Richardson W, Hey L. Reliability, validity, and responsiveness of the short form 12-item survey (SF-12) in patients with back pain. Spine. 2003. https://doi.org/10.1097/01.BRS.0000083169.58671.96.

63. Roland M, Morris R. A study of the natural history of back pain Part I: development of a reliable and sensitive measure of disability in low-back pain. Spine. 1983. https://doi.org/10.1097/00007632-198303000-00004.

64. Bellamy N, Buchanan WW, Goldsmith CH, Campbell J, Stitt LW. Validation study of WOMAC: a health status instrument for measuring clinically important patient relevant outcomes to anti-rheumatic drug therapy in patients with osteoarthritis of the hip or knee. J Rheumatol. 1988;15:1833–40.

65. Roos EM, Klassbo M, Lohmander LS. WOMAC osteoarthritis index. Reliability, validity, and responsiveness in patients with arthroscopically assessed osteoarthritis. Western Ontario and MacMaster universities. Scand J Rheumatol. 1999;28:210–5.

66. McCaffery M, Beebe A. Pain: clinical manual for nursing practice. Baltimore: V.V. Mosby Company; 1989.

67. van Tubergen A, Debats I, Ryser L, Londoño J, Burgos-Vargas R, Cardiel MH, Landewé R, Stucki G, Van Der Heijde D. Use of a numerical rating scale as an answer modality in ankylosing spondylitis–specific questionnaires. Arthritis Rheum. 2002. https://doi.org/10.1002/art.10397.

68. Dunn KM, Croft PR. Classification of low back pain in primary care: using "bothersomeness" to identify the most severe cases. Spine. 2005;30:1887–92.

69. Zigmond AS, Snaith RP. The hospital anxiety and depression scale. Acta Psychiatr Scand. 1983. https://doi.org/10.1111/j.1600-0447.1983.tb09716.x.

70. Montazeri A, Vahdaninia M, Ebrahimi M, Jarvandi S. The hospital anxiety and depression scale HADS): translation and validation study of the Iranian version. Health Qual Life Outcomes. 2003. https://doi.org/10.1186/1477-7525-1-14.

71. Group TE. EuroQol-a new facility for the measurement of health-related quality of life. Health Policy. 1990;16:199–208.

72. Hurst NP, Kind P, Ruta D, Hunter M, Stubbings A. Measuring health-related quality of life in rheumatoid arthritis: validity, responsiveness and reliability of EuroQol (EQ-5D). Brit J Rheumatol. 1997;36:551–9.

73. Fischer D, Stewart AL, Bloch DA, Lorig K, Laurent D, Holman H. Capturing the patient’s view of change as a clinical outcome measure. JAMA. 1999. https://doi.org/10.1001/jama.282.12.1157.

74. Kamper SJ, Ostelo RW, Knol DL, Maher CG, de Vet HC, Hancock MJ. Global perceived effect scales provided reliable assessments of health transition in people with musculoskeletal disorders, but ratings are strongly influenced by current status. J Clin Epidemiol. 2010. https://doi.org/10.1016/j.jclinepi.2009.09.009.

75. Williams GC, Freedman ZR, Deci EL. Supporting autonomy to motivate patients with diabetes for glucose control. Diabetes Care. 1998. https://doi.org/10.2337/diacare.21.10.1644.

76. Brooks JM, Kaya C, Chan F, Thompson K, Sanchez J, Parker Cotton B, Fortuna K. Validation of the Behavioral Regulation in Exercise Questionnaire-2 (BREQ-2) for adults with chornic musculoskeletal disease. Int J Ther Rehab. 2018. https://doi.org/10.12968/ijtr.2018.25.8.395.

77. Williams GC, Deci EL. Internalization of biopsychosocial values by medical students: a test of self-determination theory. J Pers Soc Psychol. 1996. https://doi.org/10.1037/0022-3514.70.4.767.

78. Mullan E, Markland D, Ingledew DK. A graded conceptualisation of selfdetermination in the regulation of exercise behaviour: development of a measure using confirmatory factor analytic procedures. Pers Individ Differ. 1997;23(5):745–52.

79. Sullivan MJ, Bishop SR, Pivik J. The pain catastrophizing scale: development and validation. Psychol Assess. 1995. https://doi.org/10.1037/1040-3590.7.4.524.

80. Osman A, Barrios FX, Gutierrez PM, Kopper BA, Merrifield T, Grittmann L. The pain catastrophizing scale: further psychometric evaluation with adult samples. J Behav Med. 2000. https://doi.org/10.1023/A:1005548801037.

81. Lundberg MK, Styf J, Carlsson SG. A psychometric evaluation of the Tampa scale for Kinesiophobia—from a physiotherapeutic perspective. Physiother Theory Pract. 2004. https://doi.org/10.1080/09593980490453002.

82. Vlaeyen JW, Kole-Snijders AM, Boeren RG, van Eek H. Fear of movement/(re) injury in chronic low back pain and its relation to behavioral performance. Pain. 1995. https://doi.org/10.1016/0304-3959(94)00279-N.

83. Craig CL, Marshall AL, Sjostrom M, Bauman AE, Booth ML, Ainsworth BE, Pratt M, Ekelund U, Yngve A, Sallis JF, Oja P. International physical activity questionnaire: 12-country reliability and validity. Med Sci Sports Exerc. 2003. https://doi.org/10.1249/01.MSS.0000078924.61453.FB.

84. Chisholm D, Knapp MR, Knudsen HC, Amaddeo F, Gaite L, van Wijngaarden B. Client socio-demographic and service receipt inventory-European Version: development of an instrument for international research. EPSILON Study 5.European psychiatric services: inputs linked to outcome domains and needs. Br J Psychiatry Suppl. 2000;(39):s28–33.

85. Patel A, Rendu A, Moran P, Leese M, Mann A, Knapp M. A comparison of two methods of collecting economic data in primary care. Fam Pract. 2005. https://doi.org/10.1093/fampra/cmi027.
