# Supplementary material for: Feasibility cluster randomised controlled trial evaluating a theory-driven group-based complex intervention versus usual physiotherapy to support self-management of osteoarthritis and low back pain (SOLAS)
Source: Trials. 2020 Sep 23;21:807. doi: 10.1186/s13063-020-04671-x (PMC7510107; doi:10.1186/s13063-020-04671-x)
Supplement: Supplementary file 3 — Additional file 3. Qualitative Interview Methods and Results. [file 13063_2020_4671_MOESM3_ESM.docx]

**Additional file 3: Qualitative Interviews**

Analysis of interview data was an iterative process which involved reading the transcripts and identifying recurrent patterns or themes. A list of codes was generated that were grouped together to develop a list of themes and sub-themes, which were reviewed to ensure relevance to the theme. The transcripts were then re-read to ensure all data was accounted for during initial coding and analysis. Finally, theme and sub-theme names were refined, and the identified themes were subsequently mapped to the relevant feasibility criteria. Using the relevant coding frame, independent raters’ (DH, JM) independently double coded sample data (25%) from both participant and PT interviews with 70% agreement taken as the minimum cut-off rate of agreement [86].

**Acceptability of the SOLAS intervention and trial procedures to participants and physiotherapists**

| **Definitions** | **Participant interviews** | **Physiotherapist interviews** | | |
| --- | --- | --- | --- | --- |
| **Acceptability of SOLAS intervention** | Defined as the extent to which participants who have received the SOLAS intervention consider the content, mode of delivery and support materials acceptable, appropriate, and satisfactory in meeting their needs | | Defined as the extent to which physiotherapists who have delivered the SOLAS intervention consider the training, content, mode of delivery and support materials acceptable and appropriate in meeting their needs and those of their patients within their service context | |
| **Themes and number of individuals raising each theme** | **Sample quotes with ID numbers** | | | |
| Impressions of the intervention content and mode of delivery  *(Participants n=12 ;*  *PTs n=11)*  Positive aspects of the intervention  *(Participants n=12;*  *PTs n=11)*  Mixed group involved  *(PTs n=11)* | *‘I just felt it worked on every level really and was a very very positive experience and I'd highly recommend it… I was so wrong to think for a minute that individual was in any way superior. Ok I didn't get to do it so I've never really worked with a physiotherapist one-to-one. But whatever we did it really worked and it was really good, and so I'm a big fan now.*’ [ID44]  ‘*You just get to hear from other people’s problems, and how they cope and what they do. And, some of them might be relevant to yourself, you know because other people had different sort of problems, you know, knee problems and things and not necessarily the back problem…I always find it interesting, you know, to listen to what other people say and what their experiences are*’ [ID02] | *‘they enjoyed it a huge amount, absolutely. I think the general feedback was that everybody got something out of it without a doubt*’. [P6] | | *‘in primary care, you know, a lot of our initiatives are around setting up new groups in the area, emmm, in our kind of local areas … you know the SOLAS group would be something that we’d like to kind of…keep on … it would be something that we could certainly roll out in the community’.* [P3]  *‘The classes seem to be the most kind of appropriate and best way to use our time’.* [P8]  ‘*I think it works I mean- certainly arthritis and back pains are big ones but it could be delivered to others as well’*. [P5]  *‘therapists on the ground are going to have to become familiar with the style of- it's a different style of- of providing physio…But we're you know maybe we're traditionally taught about you know they come in we tell them what to do, they go home they're supposed to do it. Emm this is slightly different which is great news’.* [P5]  *new approach to doing a group setting…trying to implement the behavioural change strategies’*… *‘did enjoy doing it’.* [P7]  ‘*the content of the classes its obviously excellent… the education component was very straightforward and very easy to deliver…I mean so much so I would be loving to use it in the future*’ [P6]  ‘*I suppose even in a bigger group you would sort of be giving everyone individual attention and-and that. I seem to get quite positive feedback*’. [P7]  ‘*the information that you're giving on self-management for OA and back pain is very similar’* and .. ‘previously *looked into a more multi-joint intervention as opposed to just focusing on back pain*’ *the SOLAS intervention* *came at a very good time’.* [P7] |
| Impressions of the intervention resources  *(Participants n=12;*  *PTs n=11)* | ‘…*the pedometer has helped me a lot in that it gets me moving. You know, so, that's a positive*.’ [ID62]  ‘*Yeah I refer back to the booklet as well, yeah…Am, it would vary maybe once or twice a week maybe’* [ID06]  *‘The meals in the cookbook I definitely find them good.’* [ID01]  *‘I had the CD on there, and I listening to it. Eh it's like a hypnotic effect, listening to it…It calms you, and it teaches you to be mindful of where you are , the position you're in, do you know what I mean?..it's a powerful thing.’* [ID43] | ‘..*nothing but positive feedback for all the content emm and the-the resources*.’ [P6]  *‘…emmm, the pedometers were a real hit!’*[P2]  *‘some of the other ladies really felt that they could oh I did much better this week look I’m after walking further or I'm after you know doing x, y and z without the same amount of pain or I didn't take or have to take any medication this week so I think they found the diary ehh very helpful to reflect back on that.’* [P10] | | *Emm I mean if anything I'd be trying to incorporate the resources into what I was doing before. Emm so I mean I just think they complimented the-the education fantastically I-I just thought they added much more to the program than not having these resources‘* [P6]  *‘The patient manual and the pedometer, the relaxation CD. etcetera I thought it was very well put-together yeah*.’[P11]  *‘I think the actions plans emm I think they're good and obviously we need to encourage them.’* [P5] |
| Benefits for Participants  *Physical*  *(Participants n=7 ;*  *PTs n=10)*  *Psychological*  *(Participants n=3 ;*  *PTs n=8)*  *Social*  *(Participants n= 8;*  *PTs n=7)* | *‘…the physiotherapy that I done must have helped and the little things that were going on there like, I mean, obviously my knee improved from going there, so I don't know it didn't improve from anything else… And I managed the pain a little bit better.’* [ID67]  *‘It's the specific exercises for my knee that I would have learned. And maybe it just confirmed for me, you know, that I was going the right way…Which I thought that was useful, because sometimes you don't know, em, like, if I go out there and walk am I doing more damage?...And that confirmed for me that no I wasn't going to do more damage.’* [ID06]  *‘I have a different mindset if you like…that you can cope, like beforehand you might have thought oh I can't cope with this, whereas now I feel I can cope with it…Well, just saying to yourself that you're not doing any damage by doing it*…*I think people in general might have a fear that if you feel pain that you should just stop. Well you do stop and have a rest but, keep going again …that was the one thing that I got, one big thing I felt I got out of the programme…I would have thought beforehand that it, you would be damaging if you did something. I don't feel that now.*’ [ID01]  *‘…as I said you'd know that someone else was in the same boat as you, maybe a different pain, eh, understanding other people's form of pain as well as your own.’* [ID17] | ‘*she got a couple of specific strengthening exercises for her knee which gave her huge relief’.* [P2]  *‘ ….by the time I was leaving he said he had much less pain’. [P11]*  ‘*I think it's empowered and skilled people as much as possible in order to be you know to be independent and-and to self-manage their condition’*. [P6]  ‘*Well I suppose the decrease in fear was the big thing’.* [P11]  *It gave a lot of opportunity for people to interact and give their own opinions and just air whatever their concerns were and just again to have other people listen to them.* [P8]  ‘*yes they were getting out and exercising more but they were socialising more, they were going out more. They were able to, you know, get the Luas into town and…do things they hadn’t done in years … They were more socially kind of active as well as actually physically aerobically active*’. [P4] | |  |
| Self-management promotion  *(Participants n=11 ;*  *PTs n=11)* | *‘It would have a very good effect. If I'm in pain, if my knee is bothering me and I go out for a walk, within about two kilometers, the pain is almost gone.’* [ID06]  ‘*I’d say the aim for myself was to be more in control really of my pain, and of myself. You know, and em..not just think that well this is how I am, and this is how it’s going to stay, but to give me, I suppose, the power with my own body and that it helped with that..You know, so that I don’t just hand my body over to a hospital and say ‘fix me’* [ID62] |  | | *‘We're going to give them I suppose the skills necessary so that they will be able to manage their pain more efficiently by understanding what their condition and giving them skills that they needed you know to self-manage you know and each week you know that was explained to them so by the end yeah I think it's you know it's it definitely did emm do-what it's supposed’. [P7]* |
| Impressions of PT training programme  *(PTs n=10)* |  |  | | *‘got across to me hugely the self-management aspect’* [P2] |
| **Acceptability of trial recruitment procedures** | Defined as the extent to which participants consider taking part in the trial, follow-ups, and outcome measure completion acceptable and appropriate | | | Defined as the extent to which physiotherapists who have participated in the trial consider trial recruitment procedures acceptable and appropriate |
| Experience of involvement in research project  *(Participants n=9;*  *PTs n=11)* | *I was grateful of the opportunity to go and experience that like, yeah I, you know it was good to be part of that, the study… So I hope it's helping other people’.* [ID 05]  *‘well done for everything and thank you for these calls and thank you for your time, you and your colleagues for being interested in us*…*I really do feel I'm better. You know what was actually quite nice that I knew in the future there would be a follow up call or whatever. It was quite nice in a way, you always like to think that someone is there, keeping half an eye out’.* [ID 02] | | | ‘*I think it's great to see emm work being done in this area and-and I think we just need to keep very flexible I mean it's-it's-it's an expanding area. There's stuff coming out all the time and not to- not to get fixed on “ok this is the accurate programme’.* [P5]  ‘*they were very prompt and any queries that I had you know were dealt with you know in a timely matter*’. [P7] |

**Physiotherapist views of the feasibility of the SOLAS intervention and trial recruitment procedures**

| **Feasibility criteria** | **Feasibility Definitions** | |
| --- | --- | --- |
| **Demand of SOLAS intervention** | The extent to which physiotherapists perceive the demand and positive/negative effects of participating in training, studying intervention materials, preparing class venue and delivering the SOLAS intervention, using specified behaviour change strategies | |
| **Themes and number of PTs raising each theme** | **Sub-themes** | **Sample quotes and physiotherapist ID numbers** |
| Challenges to delivery | Volume of content  *(PTs n=5)* | *‘I found the content in week 1 was nearly too much…very packed as in by the time I finished talking and ran through the exercises the hour and a half was finished. And so nobody actually practiced any of the exercises on-the first day’.* [P10] |
|  | Overemphasis on goal setting  *(PTs n=4)* | *‘I maybe found you know when those participants were engaging with the exercise diary I felt like every week those slides came up again with the setting your goals, what are your aims what are your you know and that-became a little repetitive*’. [P 10] |
|  | Language use  *(PTs n=5)* | *‘..whereas actually as the weeks go on you find that you-you do you know and I suppose there's-there's a lot as well that the client takes-takes more control that you don't need to do as much.’ [P6]*  ‘*trying to steer away from that controlling behaviour but at the same time, you know, you kinda do want to give them feedback but … that balance can be difficult at times’*. [P3] |
|  | Use multiple exercise stations  *(PTs n=3)* | *“I tried to set up dedicated mats for too many of the exercises that was making it, making the whole process maybe complicated for patients.”* [P1]  *‘so I’d probably just have one pod for each and they’d go there and they can try out some of the hip exercises, and similarly then for the knee’.* [P5] |
| Class size  *(PTs n=11)* | *‘I felt during the exercise component that if there were more people in the class I found that at times I was kind of being kind of quiet cause I'd already addressed kind of goals with people when they were on the machines and doing their exercises…So there was a little silence in the class when they were off exercising by themselves whereas if there were maybe 6 or 7 or even 8 people in the class you could have gone round to each person individually and the whole 45 minute session would have been taken up by me going round to everybody.’* *’ [P8]* | |
| Attendance  *(PTs n=10)* | *‘…if someone missed a week then you were trying to catch ehh you know trying to help them catch up. So I found that a little bit challenging…. There was 5 in the group but only 2 finished in the last week. So I think they might have missed out a little bit on a group dynamic as such.’* [P7] | |
| Time Involved  *(PTs n=11)* | *‘it does demand more because you're- you have to do a lot more preparatory work like you know before each talk I would have had to sit down read the material and familiarise myself with it but that's the same with every programme I think-with every new programme so I wouldn't say maybe that was necessarily different’.* [P7]  ‘*the classes that I’d done previously were a little bit easier to deliver. They weren’t…like yea...the sessions that I was familiar with, the education content was easier due to more familiarity with it*… *the experience was a lot more positive’* [P1] | |
| Level of Engagement  *(PTs n=11)* | *‘They seemed to be engaging in between the weeks and there was something maybe I was trying to bring up about the barriers and you know the barriers during the week but actually in fact I don't know I just have a really good cohort but they seem to all yes there were challenges by communions and weather and that sort of thing but they seemed to-to come to overcome those barriers and they're working fine during the week.’* [P6]  ‘*‘there was one emm there was one client and he might have said a few times that ah sure I've done enough now and he kind of skidadled off a bit early…Well I didn't hold him back…No I didn't. He just said “ah look it I walked down already, I've done plenty and these in that I've done a few stations here now I'll be grand”…So you know I just had to respect that I guess.’* [P4] | |
| Literacy  *(PTs n=3)* | *‘some of the information might have a little bit technical for the patient group that we're dealing with but I mean you can always tweak that depending on the patients group. I suppose just more some of it might have been more medical the-the terminology and I just felt that not in every slide but just a couple of slides maybe could have been simplified a bit more’.* [P7]  *“you know and-and there are a lot of older people out there who-who would've dropped out at school at 12 or 13 and they don't like those sort of emm- writing a- homework kind of stuff, do you know what I mean?. So I tend to be very eh careful about pushing it out really…And I'm aware that-that- I suppose particularly there are a lot of patients out there and I don't want to make it awkward for anybody”* [P5].  *“Where I work there is a lot of people where literacy is very low. I've found one or few people who don't ehh- she said to me “oh I can't read you know there's no point in giving me information.”. Emm so you do come across that. I come across a lot of people where English is not their first language so therefore that’s a challenge for them”.* [P5] | |
| Feedback during Training  *(PTs n=11)* | ‘I *kind of…maybe wasn’t…totally clear on the feedback. Well I kinda got the general jist, but wasn’t 100% clear as to… I can’t remember exactly what it was, but whatever the feedback was, I wasn’t sure maybe how to…go about it differently*’. [P3] | |
| **Demand of Trial Recruitment Procedures** | The extent to which physiotherapists perceive the demand of completing their required tasks for participating in the trial. | |
| Recruitment and screening process  *(PTs n=5)* | ‘*people who were selected definitely bought in quite very well. Emm they were certainly committed to the programme’*. [P7]  *‘I mean screening was hard, it was hard to get the people who meet the criteria…*[P5]  *‘patients weren't appropriate for when they were recruiting’.* [P4]  ‘*I know for the SOLAS program we were restricted within the Swords area so it may have been that the- you know it could be opened out to a wider population which is what we do with our back class*’. [P6]  *‘the letter for instance that went out to take part in the research trial. Sure people couldn’t understand that’. [P5]*  *‘If I saw them maybe twice or three times and they know who I am and then I suggest oh I run a class-you know and it could be great for you that I thought well actually the buy in was much better than if I just met them for the first time and suggest said oh this is what I think is good for you. So a bit maybe to build up a bit more trust in them I think people still have the idea that the-the classes are the you know you got the-the raw deal’.* [P6] | |
| Knowledge of participants  *(PTs n=6)* | ‘*you feel like you’re almost going in a little bit blind at the start. Again you’re starting an intervention without having done any assessments with them, which is maybe a foreign process. So there’s that certain bit of awkwardness in the first session’*. [P1]  *‘process is just a bit easier when you know them*’. [P4] | |
| **Practicality of SOLAS Intervention Delivery** | The factors influencing the implementation of the SOLAS intervention in a range of HSE settings by a range of physiotherapists taking into account variations in staffing, facilities, equipment and class size | |
| Facilities, Equipment and Resources  *(PTs n=11)* | ‘*we had loads, all the facilities we needed really*’ [P6]. | |
| Programme Duration  *(PTs n=4)* | *‘you know people in pain it's very hard for them to commit to this type of program and to be able to sit for 45 minutes and do exercise for 45 minutes. You know some people need more time’.* [P5]  *‘feedback I got from a couple of individuals was time-wise it was a bit too lengthy’.* [P8]  *‘I always went over time because I let them talk a lot amongst themselves and then they kind of they went off- not off track but they were they were discussing very appropriate things”,* however*, ‘there were some classes there that went too long- the education part went onto an hour’.* [P6] | |
| Class Size  *(PTs n=9)* | “*we were struggling with it before emm and I don't know whether they you know you I suppose looked into why the participant number was so low but this I know these queries we were having of- how can we actually get people to-to engage- in in the programmes*”. [P5]  ‘*without numbers so I suppose that's where the feasibility comes down to. If we can't increase the participant number, it just it wouldn't be cost-effective’.* [P6]  *‘maybe 6 would be an ideal number’*. [P8] | |
| Training Location and Time  *(PTs n=9)* | ‘*kind of two full days of training, that needs to be factored into the efficiency…the time efficiency of the programme overall*.’[P3] | |
| **Adaptation of SOLAS Intervention** | The extent to which the SOLAS intervention training programme, content, mode of delivery and support materials will need to be modified during/at end of the trial to enhance its acceptability and implementation for a future definitive trial | |
| Programme Changes Made  *(PTs n=3)* | *‘…being a bit more specific with technical terms with people who understand emm have maybe a better understanding.*’ [P9] | |
| Suggested Programme Changes  *(PTs n=8)* | *‘I found the content in week 1 was nearly too much…very packed as in by the time I finished talking and ran through the exercises the hour and a half was finished. And so nobody actually practiced any of the exercises on the first day…if there was anything to change it was it might be just the content on how it was loaded in the beginning.’* [P10]  *‘So maybe just to shorten it to you know 20 minutes, 25…The presentation bit.’* [P9]  ‘*No you've got to kinda keep things so simple, so I keep going back to the health literacy level. You've got to you know and even fill the booklets. There's quite a lot of words in it you know. I think keep working on the visuals. Keep streamlining those…even you know busy people or well-educated people don’t sit down and read things in detail either*.’ [P5]  *‘I would recommend you'd do it the other way around. Yeah. Cause if you get people in and they can be doing a bit of exercise and you could be having a bit of chat and it's a bit more informal and they get warmed up and they get going and you get the emm endorphins release to get everything going and then you bring them in for the chat and you'll get much more out of them.’* [P5] | |
| Suggested Changes to Training  *(PTs n=7)* | *‘‘…I would struggle with is the kind of psychological component and the approach. Emm so maybe a bit of a recap in terms of the delivery cause I suppose it’s easy to fall back into- maybe we'll prescribe an exercise again. So that might be an issue’.* [P9]  “*more scenarios of challenging participants*” [P5] | |
| **Adaptation of Trial recruitment procedures** | The extent to which trial recruitment procedures, including physiotherapists tasks, will need to be modified during/at end of the trial to enhance acceptability and implementation for a future definitive trial | |
| Suggested Changes to Trial  *(PTs n=6)*  Recruitment Procedures  *(PTs n=5)* | *‘Perhaps a standardized you know summary sheet could be done up so you know that information is given to the physiotherapist’.* [P6]  *‘So maybe to start with bigger groups but maybe it wouldn't in- with the space that we have here it wouldn't be feasible but if it was possible’.* [P9] | |

**Reference**

86. Guerin S, Hennessy E. Pupils’ definitions of bullying. Eur J Psychol Educ. 2002. https://doi.org/10.1007/BF03173535.
