# Supplementary material for: Feasibility cluster randomised controlled trial evaluating a theory-driven group-based complex intervention versus usual physiotherapy to support self-management of osteoarthritis and low back pain (SOLAS)
Source: Trials. 2020 Sep 23;21:807. doi: 10.1186/s13063-020-04671-x (PMC7510107; doi:10.1186/s13063-020-04671-x)
Supplement: Supplementary file 4 — Additional file 4. Physiotherapist Baseline Characteristics. [file 13063_2020_4671_MOESM4_ESM.docx]

**Additional file 4: Physiotherapist Baseline Characteristics**

| **Physiotherapist Characteristics** | **SOLAS Intervention**  **(n=11)** | **Usual Physiotherapy**  **(n=13)** |
| --- | --- | --- |
| Female sex, n (%) | 10 (91%) | 11 (85%) |
| Age (years), median (IQR), | 34 (12) | 31 (9) |
| Clinical experience (years), median (IQR), | 11 (13) | 8 (9) |
| Primary care experience (years), median (IQR) | 4 (4) | 4 (4) |
| Postgraduate education, n (%) | 4 (36%) | 8 (62%) |
| Postgraduate degree awarded, n (%) | 1 (9%) | 7 (54%) |
| Previous CONNECT* training, n (%) | 6 (55%) | 4 (31%) |
| Motivational Interview training, n (%) | 4 (36%) | 2 (15%) |
| Expectation median (IQR), (0-10)   - Individual physiotherapy for CLBP | 7 (2) | 8 (1) |
| - Individual physiotherapy for OA | 7 (3) | 8 (2) |
| - Group programme for LBP | 8 (1) | 9 (2) |
| - Group-programme for OA | 8 (2) | 9 (3) |
| General Causality Orientations Scale~ |  |  |
| - Autonomous orientation, median (IQR) | 100 (9) | 101 (8) |
| - Controlling orientation, median (IQR) | 56 (20) | 56 (12) |
| - Impersonal orientation, median (IQR) | 52 (16) | 46 (14) |

*CONNECT: a self-determination theory-based communication skills training intervention - Communication Style and Exercise Compliance in Physiotherapy [29]

~General Causality Orientations Scale [87]

This scale assesses the strength of three different motivational orientations within an individual. These orientations [i.e. Autonomy, Controlled, and Impersonal], are understood as relatively enduring aspects of personality, and each orientation is theorised to exist within each individual to some degree. Higher scores on each subscale indicate higher amounts of the particular orientation.

**Reference**

87. Deci EL, Ryan RM. The general causality orientations scale: selfdetermination in personality. J Res Pers. 1985. https://doi.org/10.1016/0092-6566(85)90023-6.
