# Supplementary material for: Feasibility cluster randomised controlled trial evaluating a theory-driven group-based complex intervention versus usual physiotherapy to support self-management of osteoarthritis and low back pain (SOLAS)
Source: Trials. 2020 Sep 23;21:807. doi: 10.1186/s13063-020-04671-x (PMC7510107; doi:10.1186/s13063-020-04671-x)
Supplement: Supplementary file 5 — Additional file 5. SOLAS Intervention Sites, Physiotherapists and Participants. [file 13063_2020_4671_MOESM5_ESM.docx]

**Additional file 5: SOLAS Intervention Sites, Physiotherapists and Participants**

| **Wave** | **Site code** | **Class Size** | **Class**  **Venue** | **Physiotherapist**  **ID** | **Participant Characteristics** |
| --- | --- | --- | --- | --- | --- |
| 1 | A2 | 2 | PCCC clinic | P1* | F, 70 years, knee pain  M, 58 years, knee pain |
| 1 | B1 | 6 | PCCC clinic | P2 | F, 59 years, back pain  F, 70 years, back pain  F, 79 years, hip & knee pain  F, 62 years, knee pain  F, 82 years, hip and back pain  F, 74 years, knee pain |
| 1 | C1 | 4 | PCCC clinic | P3 | F, 66 years, back & knee pain  F, 77 years, hip, knee & back pain  F, 87 years, knee pain  F, 50 years, back pain |
| 1 | D1 | 4 | Local community gym | P4 | F, 59 years, back pain  F, 67 years, hip and knee pain  M, 83 years, knee pain  F, 72 years, back & knee pain |
| Mean |  | 4.0 |  |  |  |
| 2 | E2 | 6 | Local  community gym | P5* | M, 55 years, back pain  M, 65 years, knee pain  M, 56 years, knee pain  F, 46 years, back pain  F, 68 years, back pain  F, 48 years, back pain |
| 2 | F1 | 3 | Local community centre | P6 | M, 45 years, back pain  F, 51 years, back pain  M, 34 years, back pain |
| 2 | G1 | 4 | PCCC clinic | P7 | F, 64 years, knee pain  M, 40 years, back pain  F, 84 years, back pain  M, 61 years, hip, knee, back pain |
| 2 | D2 | 4 | Local community gym | P8 | M, 45 years, back pain  F, 84 years, back & knee pain  F, 79 years, hip, knee & back pain  M, 62 years, back pain |
| 2 | C2 | 3 | PCCC clinic | P3* | M, 54 years, knee pain  M, 59 years, hip & knee pain  F, 48 years, knee pain |
| 2 | B2 | 4 | PCCC clinic | P9 | M, 79 years, knee pain  F, 63 years, knee pain  F, 33 years, back pain  F, 44 years, back pain |
| Mean |  | 4.0 |  |  |  |
| 3 | F2 | 4 | Local community centre | P10 | F, 66 years, knee pain  M, 64 years, knee pain  M, 65 years, back pain  F, 49 years, hip pain |
| 3 | G2 | 5 | PCCC clinic | P11 | M, 59 years, back & knee pain  M, 52 years, hip & back pain  M, 65 years, back pain  F, 55 years, knee pain  M, 49 years, hip pain |
| Mean |  | 4.5 |  |  |  |
|  |  |  |  |  |  |
|  |  |  |  |  |  |

* Intervention delivered for second time by an individual physiotherapist
