# Supplementary material for: Feasibility cluster randomised controlled trial evaluating a theory-driven group-based complex intervention versus usual physiotherapy to support self-management of osteoarthritis and low back pain (SOLAS)
Source: Trials. 2020 Sep 23;21:807. doi: 10.1186/s13063-020-04671-x (PMC7510107; doi:10.1186/s13063-020-04671-x)
Supplement: Supplementary file 6 — Additional file 6. Usual Physiotherapy Treatment. [file 13063_2020_4671_MOESM6_ESM.docx]

**Additional file 6: Usual Physiotherapy Treatment**

|  | **Number of participants** | **Valid percent** |
| --- | --- | --- |
| Advice   - Pathophysiology - Expected course - Pain - ADL or work - Posture - Weight or Diet - Heat or Ice - TENS - General activity - Relaxation - Other* | 44  39  40  28  34  22  30  1  46  11  32 | 82  72  74  52  63  41  56  2  85  20  59 |
| Exercise – in treatment   - Core stability - Strength - Flexibility - Aerobic | 27  42  41  18 | 50  78  76  33 |
| Psychologically-informed   - Cognitive behavioural therapy - Other^#^ | 7  3 | 13  6 |
| Other   - Manual therapy - Electrotherapy - Acupuncture | 31  6  5 | 57  11  9 |
| Home programme   - Prescribed exercise - Walking programme - Other^ϕ^ | 53  39  22 | 98  72  41 |

Advice other*=footwear, sleep, breathing, smoking cessation, Psychologically-informed other^#^ = stress management, reassurance, distraction therapy, Home programme other^ϕ^=swimming, gait, pilates, cycling.
