# Supplementary material for: Feasibility cluster randomised controlled trial evaluating a theory-driven group-based complex intervention versus usual physiotherapy to support self-management of osteoarthritis and low back pain (SOLAS)
Source: Trials. 2020 Sep 23;21:807. doi: 10.1186/s13063-020-04671-x (PMC7510107; doi:10.1186/s13063-020-04671-x)
Supplement: Supplementary file 7 — Additional file 7. Methods of Follow-up. [file 13063_2020_4671_MOESM7_ESM.docx]

**Additional file 7: Methods of Follow-up**

|  | **Methods of Follow-up** | | | | | |  |
| --- | --- | --- | --- | --- | --- | --- | --- |
|  | **Post** | | | **Phone** | | | **Total** |
| **Time point** | **SOLAS**  **Intervention** | **Usual**  **PT** | Subtotal | **SOLAS Intervention** | **Usual PT** | Subtotal |  |
| **6 weeks**  **(14 pages)** | 3  (3%) | 10  (10%) | 13  (13%) | 45  (46%) | 39 (40%) | 84 (87%) | **97** (100%) |
| **2 months** |  |  |  |  |  |  |  |
| Full questionnaire  (19 pages) | 9  (9%) | 14  (15%) | 23  (24%) | 38  (40%) | 35 (37%) | 73 (76%) | 96 (100%) |
| Brief questionnaire | 1  (20%) | 2  (40%) | 3  (60%) | 2  (20%) | 0  (0%) | 2  (40%) | 5  (100%) |
| Total  responses |  |  |  |  |  |  | **101** |
| **6 months** |  |  |  |  |  |  |  |
| Full questionnaire  (27 pages) | 8  (10%) | 13  (16%) | 21  (26%) | 31  (38%) | 30 (37%) | 61 (74%) | 82 (100%) |
| Brief questionnaire  (17 pages) | 0  (0%) | 4  (100%) | 4  (100%) | 0  (0%) | 0  (0%) | 0  (0%) | 4  (100%) |
| Total responses |  |  |  |  |  |  | **86** |
| **Total** | 21 | 43 | 64  (23%) | 116 | 104 | 220 (78%) | 284 (100%) |
