# Supplementary material for: Feasibility cluster randomised controlled trial evaluating a theory-driven group-based complex intervention versus usual physiotherapy to support self-management of osteoarthritis and low back pain (SOLAS)
Source: Trials. 2020 Sep 23;21:807. doi: 10.1186/s13063-020-04671-x (PMC7510107; doi:10.1186/s13063-020-04671-x)
Supplement: Supplementary file 8 — Additional file 8. Consolidated Criteria for Reporting Qualitative Research (COREQ) Guidelines for Physiotherapist and Participant interviews. [file 13063_2020_4671_MOESM8_ESM.docx]

**Additional file 8: Consolidated Criteria for Reporting Qualitative Research (COREQ) Guidelines for Physiotherapist and Participant interviews**

**Physiotherapist Interviews**

| Reporting Item. | Item No. | Description |
| --- | --- | --- |
| **Domain 1: Research team and reflexivity** | |  |
| ***Personal characteristics***  Interviewer/facilitator  Credentials  Occupation  Gender  Experience and Training  ***Relationship with participants***  Relationship established  Participant knowledge of the interviewer  Interviewer Characteristics | 1  2  3  4  5  6  7  8 | Suzanne Guerin (SG)  PhD  Psychologist  Female  Interviewer has over 22 years’ experience in qualitative research techniques.  No prior relationship established.  PTs were provided with a participant information leaflet during the recruitment phase of the study which explained the reasons for conducting the study. No specific information about the interviewer was provided to participants.  SG was identified as a co-investigator and member of the SOLAS research team with an interest in exploring PTs’ experiences in relation to their involvement in the delivery of the SOLAS intervention within their service area. |
| \| **Domain 2: Study design** \| \| --- \| |  |  |
| ***Theoretical framework***  Methodological orientation and Theory  ***Participant selection***  Sampling  Method of approach  Sample size  Non-participation  ***Setting***  Setting of data collection  Presence of non-participants | 9  10  11  12  13  14  15 | A Realist/Essentialist paradigm underpinned the qualitative data analysis, with a thematic analysis approach [30] adopted to identify recurrent patterns and themes related to acceptability and feasibility of the intervention.  Eligible PTs were purposively selected by PT managers based on affiliation with suitable study sites, interest, experience and caseload.  Face-to-Face and Email  15 PTs were recruited to deliver the SOLAS intervention, of which 11 delivered it and 10 were interviewed.  One PT was unavailable for interview at the end of the study.  Telephone interviews were conducted.  No |
| Description of sample  ***Data collection***  Interview guide  Repeat Interviews  Audio/visual recording  Field notes  Duration  Data Saturation  Transcripts returned | 16  17  18  19  20  21  22  23 | PTs were working within outpatient physiotherapy PCCC clinics in Dublin/North Kildare, Ireland. PTs were selected based on their experience, level of interest and work setting.  Topic guides were developed for the physiotherapist interviews with specific questions and probes related to acceptability and feasibility of the intervention and trial recruitment procedures.  No repeat interviews took place.  All interviews were audio-recorded.  No field notes were made during or after the interviews were conducted.  22 – 50 (mins)  Data saturation was not relevant given that an exhaustive sample of participants were interviewed post intervention delivery at a single 6 month follow up time point.  Interview transcripts were not returned to the participants. |
| **Domain 3: analysis and findings**  ***Data analysis***  Number of data coders  Description of the coding tree  Derivation of themes  Software  Participant checking | 24  25  26  27  28 | Two per wave (DH coded 100% of the data & JM coded a 25% sample of data W1; DMA coded 100% of the data & JM coded a 25% sample of data W2&3)  Coding frame included (Additional file 3). W1 Inductive thematic analysis produced 19 major themes with 14 sub-themes which relate to feasibility of the intervention were identified during W1 analysis. W2&3 analysis identified 2 new themes.  W1 themes were developed iteratively through an inductive thematic analysis approach. W2&3 applied a hybrid deductive-inductive approach, using the coding frame from wave one during the deductive analysis. Wave 1 analysis identified 3 main themes related to the SOLAS Trial Procedures.  N/A  Participants did not provide feedback on the findings of this study. |
| ***Reporting***  Quotations presented  Data and findings consistent  Clarity of major themes  Clarity of minor themes | 29  30  31  32 | PT quotes were used in this paper to illustrate their experiences and views of the intervention.  Reliability of the data was confirmed at each stage of analysis, where a second researcher analyzed a random 25% sample of the data with consensus-percent reaching approximately 95% during W1 & 98% agreement reached during W2.  Major themes were clearly presented and discussed in this study in relation to feasibility criteria established.  Minor themes are also presented and discussed in the context of acceptability and feasibility of the SOLAS intervention to meet their needs and the needs of appropriate clients accessing PCCC PT services. |
|  |  |  |

**Participant Interviews**

| Reporting Item. | Item No. | Description |
| --- | --- | --- |
| **Domain 1: Research team and reflexivity** |  |  |
| ***Personal characteristics***  Interviewer/facilitator  Credentials  Occupation  Gender  Experience and Training  ***Relationship with participants***  Relationship established  Participant knowledge of the interviewer  Interviewer Characteristics  Interviewer characteristics | 1  2  3  4  5  6  7  8 | Elaine Toomey  PhD  Physiotherapist  Female  Interviewer has 5 years’ experience in qualitative research techniques.  No prior relationship established.  Participants were provided with a participant information leaflet during the recruitment phase of the study which explained the reasons for conducting the study. No specific information about the interviewer was provided to participants.  ET was identified as a co-investigator and member of the SOLAS research team. |
| \| **Domain 2: Study design** \| \| --- \| |  |  |
| ***Theoretical framework***  Methodological orientation and Theory  ***Participant selection***  Sampling  Method of approach  Sample size | 9  10  11  12 | A Realist/Essentialist paradigm underpinned the qualitative data analysis, with a thematic analysis approach [30] adopted to identify recurrent patterns and themes related to patients experiences of the SOLAS intervention.  A list of codes was identified to develop a coding frame. Relevant themes and sub-themes were mapped to feasibility criteria.  Purposive sampling  Face-to-Face and Email. Participants were identified through a multi-level approach. Following screening of PCCC PT waiting lists and consultation with referring GPs potentially eligible clients were sent an invitation letter, followed by the research team conducting a telephone and face-to-face screening and assessment to consenting participants.  12 participant interviews were analyzed in qualitative study (20% sample) |
| Non-participation  ***Setting***  Setting of data collection  Presence of non-participants  Description of sample | 13  14  15  16 | Of the 24 invited, 17 consented and 12 were interviewed  Telephone interviews were conducted in a research room in the University.  No  Patients in this study met the following criteria: chronic (≥3 months), OA working diagnosis of OA hip/knee joint: age 45 years old or over, activity-related joint pain and no morning joint-related stiffness or morning stiffness that lasts ≤30 min) [4] and/or LBP (age >=30 years old and have non-specific LBP of mechanical origin with or without radiation to the lower limb); be able to read/understand and speak English without assistance; access to a telephone for screening and available to attend a 6-week group class of 90mins/week. |
| ***Data collection***  Interview guide  Repeat Interviews  Audio/visual recording  Field notes  Duration  Data Saturation  Transcripts returned | 17  18  19  20  21  22  23 | Topic guides were developed for the participant interviews with specific questions and probes related to their views on the acceptability, demand and future adaptation to refine and improve the current intervention.  Yes – participants were interviewed 6 months post intervention. This study is based on 6 month follow up interviews.  Interviews were audio-recorded.  Field notes were made during and after the interviews were conducted.  15-40 mins  Data saturation was not relevant given that an exhaustive sample of participants were interviewed post intervention delivery.  Interview transcripts were not returned to the participants. |
| **Domain 3: analysis and findings**  ***Data analysis***  Number of data coders  Description of the coding tree  Derivation of themes  Software  Participant checking | 24  25  26  27  28 | 2 researchers coded the data (DMA coded 100% of the data and a second researcher (DH) coded a 25% random sample of the data.  Themes were developed iteratively through an inductive thematic analysis approach [30]. 3 main themes with 11 sub-themes were identified.  Themes were developed iteratively through an inductive thematic analysis approach.  N/A  Participants did not provide feedback on the findings of this study. |
| ***Reporting***  Quotations presented  Data and findings consistent  Clarity of major themes  Clarity of minor themes | 29  30  31  32 | Participant quotations were used in this paper to illustrate their experiences and views of attending the 6 week group-based physiotherapy programme.  Reliability of the data was confirmed with a second researcher analysing a random 25% sample of the data with consensus-percent reaching approximately 90%.  Major themes were clearly presented and discussed in this study in relation to feasibility criteria established.  No minor themes were identified. |
