# Supplementary material for: Feasibility cluster randomised controlled trial evaluating a theory-driven group-based complex intervention versus usual physiotherapy to support self-management of osteoarthritis and low back pain (SOLAS)
Source: Trials. 2020 Sep 23;21:807. doi: 10.1186/s13063-020-04671-x (PMC7510107; doi:10.1186/s13063-020-04671-x)
Supplement: Supplementary file 9 — Additional file 9. Baseline sociodemographic variables. [file 13063_2020_4671_MOESM9_ESM.docx]

**Additional file 9 Baseline sociodemographic variables**

| **Sociodemographic**  **variables** | **Total**  **(n=120)** | **SOLAS Intervention**  **(n= 59)** | **Usual Physiotherapy**  **(n= 61)** |
| --- | --- | --- | --- |
| Age, mean,  min-max, years | 59.0, 31-87 | 60.0, 33-87 | 57.9, 31-85 |
| Gender, n (%)  Female  Male | 77 (64)  43 (36) | 37 (63)  22 (37) | 40 (66)  21 (34) |
| BMI, mean,  min-max) kg/m^2^ | N=84  27.9, 16.6-44.9 | N=41 (18 missing)  28.6, 17.8-44.9 | N=43 (18 missing)  27.3, 16.6-44.5 |
| Diagnosis (n)  Chronic non-specific low back pain  OA hip  OA knee | 75  26  55 | 34  12  29 | 41  14  26 |
| Site(s) of pain, n (%)  Back only  Knee only  Hip only  Multi-joint, n (%)  Hip and Knee  Back and Knee  Hip and Back  Hip, Knee and Back | 52 (43)  29 (24)  8 (7)  8 (7)  13 (11)  5 (4)  5 (4) | 24 (41)  18 (31)  4 (7)  3 (5)  5 (9)  2 (3)  3 (5) | 28 (46)  11 (18)  4 (7)  5 (8)  8 (13)  3 (5)  2 (3) |
| Marital status, n (%)  Single  Married/ Cohabiting  Separated/Divorced/ Widowed | 25 (21)  57 (48)  38 (32) | 12 (20)  24 (41)  23 (39) | 13 (21)  33 (54)  15 (25) |
| Employment groups,  n (%)  Employed/ Carer  Unemployed  Employment status  Full time work  Part time work  Voluntary worker  Homemaker  Retired/ redundant  Full time carer  Unemployed  Disability | 39 (33)  81 (67)  15 (12)  16 (13)  1 (1)  14 (12)  31 (26)  7 (6)  20 (17)  16 (13) | 20 (34)  39 (66)  7 (12)  8 (14)  1 (2)  7 (12)  15 (25)  4 (6)  10 (17)  7 (12) | 19 (31)  42 (69)  8 (13)  8 (13)  0 (0)  7 (12)  16 (26)  3 (5)  10 (16)  9 (15) |
| Country of birth, n (%)  Ireland  Other | 104 (87)  16 (13) | 52 (88)  7 (12) | 52 (85)  9 (15) |
| Sick leave, n (%)  Low back pain (n=74)  Yes  No  Osteoarthritis hip or knee (n=66)  Yes  No | 35 (47)  39 (53)  9 (14)  57 (86) | N=33  13 (39)  20 (61)  N=35  6 (17)  29 (83) | N=41  22 (54)  19 (46)  N=31  3 (10)  28 (90) |
| Previous physiotherapy, n (%)  Low back pain (n=74)  Yes  No  Osteoarthritis hip or knee (n=68)  Yes  No | 39 (53)  35 (47)  12 (18)  56 (82) | N=33  16 (49)  17 (51)  N=35  7 (20)  28 (80) | N=41  23 (56)  18 (44)  N=33  5 (15)  28 (85) |
| Previous group class, n (%)  Low back pain (n=74)  Yes  No  Osteoarthritis hip or knee (n=66)  Yes  No | 6 (8)  68 (92)  2 (3)  64 (97) | N=33  3 (9)  30 (91)  N=35  0 (0)  35 (100) | N=41  3 (7)  38 (93)  N=31  2 (7)  29 (93) |
| Expectation of treatment,  mean (SD), 0-10  Individual treatment  Group treatment | 8.4 (1.8)  7.2 (2.1) | 7.3 (2.1)  7.2 (2.1) | 8.4 (1.7)  7.0 (2.1) |
| Keele STarT Back Screening Tool, mean (SD), 0-9 | 4.6 (2.2) | 4.7 (2.1) | 4.6 (2.3) |
