# Supplementary material for: Feasibility cluster randomised controlled trial evaluating a theory-driven group-based complex intervention versus usual physiotherapy to support self-management of osteoarthritis and low back pain (SOLAS)
Source: Trials. 2020 Sep 23;21:807. doi: 10.1186/s13063-020-04671-x (PMC7510107; doi:10.1186/s13063-020-04671-x)
Supplement: Supplementary file 10 — Additional file 10. Model-predicted mean (95% CI) behaviour change process model outcomes per group over time. [file 13063_2020_4671_MOESM10_ESM.docx]

**Additional file 10. Model-predicted mean (95% CI) behaviour change process model outcomes per group over time.**

| Outcome | Group | Time | | | | | | | | No. Clusters | Cluster ICC | Participant ICC |
| --- | --- | --- | --- | --- | --- | --- | --- | --- | --- | --- | --- | --- |
|  |  | Baseline | | 6 weeks | | 2 months | | 6 months | |  |  |  |
| ***Tampa Scale of Kinesiophobia (TSK-11)*** | | | | | | | | | | | | |
| Activity avoidance subscale (0-24) | Usual PT | N=61 | 16.0 (15.1, 16.9) | N=49 | 14.3 (13.4, 15.3) | N=49 | 14.3 (13.4, 15.3) | N=43 | 15.0 (14.0, 16.0) | 7 | 0.0 | 0.55 |
|  | SOLAS | N=59 | 16.0 (15.1, 16.9) | N=48 | 15.0 (14.1, 16.0) | N=45 | 14.4 (13.4, 15.3) | N=38 | 14.6 (13.6, 15.6) | 7 |  |  |
|  |  |  |  |  |  |  |  |  |  |  |  |  |
| ***Pain Catastrophising Scale (PCS)**** | | | | | | | | | | | | |
| Total score median [IQR],  (0-52)* | Usual PT | N=61 | 14.0 *(7.0, 25.0)* | N=49 | 6.0 *(2.0, 18.0)* | N=49 | 4.0 *(1.0, 13.0)* | N=43 | 2.0 *(0.0, 9.0)* | 7 | N/A | N/A |
|  | SOLAS | N=59 | 16.0 *(6.0, 34.0)* | N=48 | 9.0 *(4.0, 17.0)* | N=45 | 7.0 *(3.0, 15.0)* | N=38 | 6.0 *(2.0, 12.0)* | 7 |  |  |
| ***Behaviour Regulation Exercise Questionnaire (BREQ)*** | | | | | | | | | | | | |
| Autonomous motivation (0-16) | Usual PT | N=61 | 3.1 (2.8, 3.3) | N=49 | 3.2 (2.9, 3.4) | N=49 | 3.3 (2.9, 3.5) | N=43 | 3.1 (2.8, 3.4) | 7 | 0.21 | 0.35 |
|  | SOLAS | N=59 | 3.1 (2.8, 3.3) | N=48 | 3.4 (3.1, 3.7) | N=45 | 3.2 (2.9, 3.5) | N=39 | 3.0 (2.7, 3.3) | 7 |  |  |
| Controlled motivation (0-16) | Usual PT | N=61 | 1.1 (0.8, 1.3) | N=49 | 1.3 (1.1, 1.6) | N=49 | 1.1 (0.8, 1.3) | N=43 | 1.1 (0.8, 1.4) | 7 | 0.01 | 0.50 |
|  | SOLAS | N=59 | 1.3 (1.0, 1.6) | N=48 | 1.3 (1.1, 1.7) | N=45 | 1.1 (0.8, 1.4) | N=39 | 0.9 (0.6, 1.2) | 7 |  |  |
| Amotivation (0-8) | Usual PT | N=61 | 0.2 (0.8, 0.4) | N=49 | 0.3 (0.1, 0.5) | N=49 | 0.3 (0.1, 0.4) | N=43 | 0.4 (0.2, 0.6) | 7 | 0.14 | 0.15 |
|  | SOLAS | N=59 | 0.3 (0.8, 0.4) | N=48 | 0.3 (0.1, 0.5) | N=45 | 0.1 (-0.1, 0.3) | N=39 | 0.1 (-0.1, 0.3) | 7 |  |  |
| Relative Autonomy Index [RAI] (-12 to 32) | Usual PT | N=61 | 4.6 (3.8, 5.4) | N=49 | 4.4 (3.6, 5.3) | N=49 | 4.9 (4.1, 5.8) | N=43 | 4.3 (3.4, 5.2) | 7 | 0.23 | 0.34 |
|  | SOLAS | N=59 | 4.3 (3.4, 5.1) | N=48 | 4.8 (3.9, 5.7) | N=45 | 5.0 (4.1, 5.9) | N=39 | 4.9 (3.9, 5.9) | 7 |  |  |
| ***Treatment Self-Regulation Questionnaire (TSRQ)*** | | | | | | | | | | | | |
| Autonomous motivation (3-21) | Usual PT | N=61 | 6.4 (6.1, 6.6) | N=49 | 6.1 (5.8, 6.4) | N=49 | 6.0 (5.7, 6.3) | N=43 | 5.9 (5.6, 6.2) | 7 | 0.12 | 0.39 |
|  | SOLAS | N=59 | 6.2 (5.9, 6.5) | N=47 | 6.4 (6.1, 6.7) | N=45 | 6.0 (5.7, 6.4) | N=37 | 5.8 (5.5, 6.2) | 7 |  |  |
| Controlled motivation (3-21) | Usual PT | N=61 | 3.4 (2.9, 3.8) | N=49 | 2.9 (2.5, 3.3) | N=49 | 2.7 (2.3, 3.1) | N=43 | 2.9 (2.4, 3.3) | 7 | 0.0 | 0.54 |
|  | SOLAS | N=59 | 3.4 (2.9, 3.8) | N=47 | 3.1 (2.7, 3.5) | N=45 | 2.9 (2.5, 3.4) | N=37 | 2.6 (2.1, 3.0) | 7 |  |  |
| Amotivation  (3-21) | Usual PT | N=61 | 2.8 (2.4, 3.1) | N=49 | 2.3 (1.9, 2.6) | N=49 | 2.2 (1.8, 2.6) | N=43 | 2.3 (1.8, 2.7) | 7 | 0.04 | 0.25 |
|  | SOLAS | N=59 | 2.9 (2.6, 3.3) | N=47 | 2.3(1.9, 2.7) | N=45 | 2.2 (1.8, 2.7) | N=37 | 2.4 (1.9, 2.8) | 7 |  |  |
| RAI (-2 to 18) | Usual PT | N=61 | 3.8 (2.7, 4.9) | N=49 | 4.6 (3.4, 5.8) | N=49 | 4.9 (3.7, 6.1) | N=43 | 4.4 (3.2, 5.7) | 7 | 0.0 | 0.46 |
|  | SOLAS | N=59 | 3.1 (2.7, 4.9) | N=47 | 5.0 (3.8, 6.2) | N=45 | 4.6 (3.4, 5.9) | N=37 | 4.4 (3.0, 5.7) | 7 |  |  |
| ***Perceived Competence Questionnaire (PCS)**** | | | | | | | | | | | | |
| Physical Activity median [IQR],  (4-28)* | Usual PT | N=61 | 5.8 (*4.0, 6.8*) | N=49 | 6.0 (*4.5, 7.0*) | N=49 | 6.0 (*4.3, 6.8*) | N=43 | 5.3 (*4.0, 6.8*) | 7 | N/A | N/A |
|  | SOLAS | N=59 | 5.0 (*3.8, 6.3*) | N=48 | 6.1 (*4.9, 7.0*) | N=45 | 5.5 (*4.3, 6.8*) | N=39 | 4.8 (*3.5, 6.0*) | 7 |  |  |
| Self-management median ([QR],  (4-28)* | Usual PT | N=61 | 5.0 (*3.5, 7.0*) | N=49 | 5.0 (*4.0, 6.8*) | N=49 | 5.5 (*4.0, 6.8*) | N=43 | 6.0 (*4.0, 6.8*) | 7 | N/A | N/A |
|  | SOLAS | N=59 | 5.0 (*4.0, 6.3*) | N=48 | 5.6 (*5.0, 6.8*) | N=45 | 5.5 (*4.0, 7.0*) | N=39 | 4.8 (*4.0, 6.5*) | 7 |  |  |

* Non-Normal distributions, descriptive statistics only presented.
