# Supplementary material for: Feasibility cluster randomised controlled trial evaluating a theory-driven group-based complex intervention versus usual physiotherapy to support self-management of osteoarthritis and low back pain (SOLAS)
Source: Trials. 2020 Sep 23;21:807. doi: 10.1186/s13063-020-04671-x (PMC7510107; doi:10.1186/s13063-020-04671-x)
Supplement: Supplementary file 11 — Additional file 11. Mean (95% CI) within and between group changes for behaviour change process outcomes. [file 13063_2020_4671_MOESM11_ESM.docx]

**Additional file 11. Mean (95% CI) within and between group changes for behaviour change process outcomes**

| Outcome | Group | Time | | |
| --- | --- | --- | --- | --- |
|  |  | 6 weeks | 2 months | 6 months |
| ***TSK-11*** | | | | |
| Activity avoidance subscale | Usual PT | -1.69 (-2.59, -0.79) | -1.67 (-2.57, -0.77) | -1.00 (-1.94, -0.06) |
|  | SOLAS | -0.97 (-1.88, -0.06) | -1.67 (-2.60, -0.73) | -1.41 (-2.39, -0.42) |
|  | Group Difference: | -0.71 (-1.99, 0.56) | -0.01 (-1.30, 1.29) | 0.40 (-0.96, 1.77) |
| ***PCS*** | |  |  |  |
| Total score | Usual PT | -5.23 (-8.03, -2.44) | -7.67 (-10.46, -4.87) | -9.12 (-12.04, -6.19) |
|  | SOLAS | -6.26 (-9.09, -3.42) | -7.42 (-10.33, -4.52) | -9.74 (-12.83, -6.66) |
|  | Group Difference: | 1.02 (-2.96, 5.00) | -0.24 (-4.27, 3.79) | -0.63 (-3.63, 4.88) |
| ***BREQ*** | | | | |
| Autonomous motivation | Usual PT | 0.12 (-0.12, 0.35) | 0.21 (-0.03, 0.44) | 0.06 (-0.19, 0.31) |
|  | SOLAS | 0.32 (0.08, 0.56) | 0.16 (-0.09, 0.40) | -0.02 (-0.28, 0.23) |
|  | Group Difference: | -0.21 (-0.54, 0.13) | 0.05 (-0.29, 0.39) | 0.08 (-0.27, 0.44) |
| Controlled motivation | Usual PT | 0.27 (0.00, 0.54) | -0.00 (-0.27, 0.27) | 0.02 (-0.25, 0.30) |
|  | SOLAS | 0.08 (-0.19, 0.35) | -0.19 (-0.46, 0.09) | -0.37 (-0.66, -0.08) |
|  | Group Difference: | 0.19 (-0.19, 0.57) | 0.19 (-0.20, 0.57) | 0.39 (-0.01, 0.79) |
| Amotivation | Usual PT | 0.04 (-0.17, 0.25) | 0.01 (-0.21, 0.22) | 0.17 (-0.05, 0.39) |
|  | SOLAS | -0.01 (-0.22, 0.21) | -0.12 (-0.34, 0.10) | -0.17 (-0.40, 0.06) |
|  | Group Difference: | 0.05 (-0.26, 0.35) | 0.13 (-0.18, 0.44) | 0.34 (0.02, 0.66) |
| RAI | Usual PT | -0.14 (-0.89, 0.61) | 0.42 (-0.34, 1.17) | -0.27 (-1.06, 0.51) |
|  | SOLAS | 0.57 (-0.19, 1.33) | 0.75 (-0.03, 1.53) | 0.66 (-0.15, 1.48) |
|  | Group Difference: | -0.71 (-1.78, 0.36) | -0.33 (-1.41, 0.75) | -0.94 (-2.07, 0.20) |
| ***TSRQ*** | | | | |
| Autonomous motivation | Usual PT | -0.29 (-0.58, -0.00) | -0.33 (-0.62, -0.04) | -0.42 (-0.73, -0.12) |
|  | SOLAS | 0.21 (-0.09, 0.50) | -0.12 (-0.42, 0.18) | -0.32 (-0.64, 0.00) |
|  | Group Difference: | -0.50 (-0.91, -0.09) | -0.20 (-0.62, 0.21) | -0.10 (-0.54, 0.34) |
| Controlled motivation | Usual PT | -0.44 (-0.85, -0.03) | -0.67 (-1.08, -0.26) | -0.49 (-0.92, -0.06) |
|  | SOLAS | -0.28 (-0.70, 0.14) | -0.44 (-0.87, -0.01) | -0.82 (-1.28, -0.36) |
|  | Group Difference: | -0.16 (-0.75, 0.43) | -0.23 (-0.83, 0.36) | 0.33 (-0.30, 0.96) |
| Amotivation | Usual PT | -0.47 (-0.94, -0.00) | -0.52 (-0.99, -0.05) | -0.49 (-0.97, 0.00) |
|  | SOLAS | -0.64 (-1.12, -0.16) | -0.70 (-1.19, -0.21) | -0.59 (-1.11, -0.07) |
|  | Group Difference: | 0.17 (-0.50, 0.84) | 0.18 (-0.50, 0.85) | 0.10 (-0.61, 0.82) |
| RAI | Usual PT | 0.78 (-0.47, 2.02) | 1.04 (-0.20, 2.29) | 0.58 (-0.72, 1.88) |
|  | SOLAS | 1.96 (0.69, 3.23) | 1.58 (0.29, 2.87) | 1.33 (-0.05, 2.71) |
|  | Group Difference: | -1.19 (-2.96, 0.59) | -0.54 (-2.33, 1.25) | -0.75 (-2.64, 1.15) |
| ***PCQ*** | |  |  |  |
| Physical Activity | Usual PT | 0.40 (-0.03, 0.84) | 0.17 (-0.26, 0.61) | -0.06 (-0.52, 0.39) |
|  | SOLAS | 0.78 (0.34, 1.22) | 0.26 (-0.19, 0.72) | -0.22 (-0.70, 0.25) |
|  | Group Difference: | -0.37 (-0.99, 0.25) | -0.09 (-0.72, 0.53) | 0.16 (-0.50, 0.82) |
| Self-management | Usual PT | 0.18 (-0.25, 0.62) | 0.38 (-0.05, 0.82) | 0.21 (-0.25, 0.66) |
|  | SOLAS | 0.64 (0.20, 1.08) | 0.40 (-0.06, 0.85) | 0.22 (-0.26, 0.69) |
|  | Group Difference: | -0.46 (-1.07, 0.16) | -0.01 (-0.64, 0.61) | -0.01 (-0.66, 0.65) |
