# Supplementary material for: Feasibility cluster randomised controlled trial evaluating a theory-driven group-based complex intervention versus usual physiotherapy to support self-management of osteoarthritis and low back pain (SOLAS)
Source: Trials. 2020 Sep 23;21:807. doi: 10.1186/s13063-020-04671-x (PMC7510107; doi:10.1186/s13063-020-04671-x)
Supplement: Supplementary file 12 — Additional file 12. Exploratory Analysis by Joint Pain Condition. [file 13063_2020_4671_MOESM12_ESM.docx]

**Additional file 12: Exploratory Analysis by Joint Pain Condition**

*Target Behaviours*

1. Physical Activity - IPAQ, mean (SD) MET-minutes/week

|  | Time | | | |
| --- | --- | --- | --- | --- |
|  | Baseline | 6 weeks | 2 months | 6 months |
| **Hip** | | | | |
| Usual PT | 2937 (3017) | 2994 (2868) | 2294 (1709) | 3020(4156) |
| SOLAS | 2478 (1826) | 1492(1216) | 1872(1246) | 1287 (1120) |
| **Knee** | | | | |
| Usual PT | 2493 (2393) | 3939(3695) | 2487(2164) | 2348(1935) |
| SOLAS | 2804 (2516) | 3036 (2733) | 3351(3074) | 1878 (1687) |
| **Back** | | | | |
| Usual PT | 2553 (2847) | 2791 (2740) | 2490 (3141) | 2972 (3533) |
| SOLAS | 2664 (2581) | 2861(2516) | 2847(2336) | 2587 (2148) |

1. Self-Management Behaviours - SMBQ

Set goals n (%)

|  | Time | | | |
| --- | --- | --- | --- | --- |
|  | Baseline | 6 weeks | 2 months | 6 months |
| **Hip** | | | | |
| Usual PT | 5 (36%) | 6 (55%) | 7 (58%) | 2 (20%) |
| SOLAS | 2 (17%) | 6 (60%) | 7 (70%) | 4 (36%) |
| **Knee** | | | | |
| Usual PT | 10 (39%) | 13 (62%) | 17 (77%) | 10 (53%) |
| SOLAS | 9 (31%) | 21 (81%) | 15 (56%) | 9 (41%) |
| **Back** | | | | |
| Usual PT | 12 (29%) | 15 (48%) | 13 (39%) | 12 (46%) |
| SOLAS | 12 (35%) | 19 (68%) | 14 (48%) | 9 (36%) |

Exercised in line with goals, n (%)

|  | Time | | | |
| --- | --- | --- | --- | --- |
|  | Baseline | 6 weeks | 2 months | 6 months |
| **Hip** | | | | |
| Usual PT | 5 (36%) | 5 (46%) | 7 (58%) | 2 (20%) |
| SOLAS | 2 (18%) | 5 (50%) | 8 (80%) | 4 (36%) |
| **Knee** | | | | |
| Usual PT | 10 (39%) | 13 (62%) | 17 (77%) | 10 (53%) |
| SOLAS | 9 (31%) | 21 (81%) | 15 (55%) | 9 (41%) |
| **Back** | | | | |
| Usual PT | 12 (29%) | 15 (48%) | 13 (39%) | 12 (46%) |
| SOLAS | 12 (35%) | 19 (68%) | 14 (48%) | 9 (36%) |

Performed small regular activity, n (%)

|  | Time | | | |
| --- | --- | --- | --- | --- |
|  | Baseline | 6 weeks | 2 months | 6 months |
| **Hip** | | | | |
| Usual PT | 13 (93%) | 11 (100%) | 12 (100%) | 8 (80%) |
| SOLAS | 10 (83%) | 8 (80%) | 10 (100%) | 9 (82%) |
| **Knee** | | | | |
| Usual PT | 25 (96%) | 21 (100%) | 21 (96%) | 18 (95%) |
| SOLAS | 21 (72%) | 23 (89%) | 26 (96%) | 20 (91%) |
| **Back** | | | | |
| Usual PT | 33 (81%) | 28 (90%) | 29 (88%) | 22 (85%) |
| SOLAS | 25 (74%) | 27 (96%) | 26 (90%) | 23 (92%) |

Used mental relaxation techniques, n (%)

|  | Time | | | |
| --- | --- | --- | --- | --- |
|  | Baseline | 6 weeks | 2 months | 6 months |
| **Hip** | | | | |
| Usual PT | 4 (29%) | 0 (0%) | 2 (17%) | 1 (10%) |
| SOLAS | 4 (33%) | 3 (30%) | 3 (30.0%) | 2 (18%) |
| **Knee** | | | | |
| Usual PT | 6 (23%) | 3 (14%) | 3 (14%) | 6 (32%) |
| SOLAS | 7 (24%) | 12 (46%) | 11 (41%) | 9 (41%) |
| **Back** | | | | |
| Usual PT | 11 (27%) | 6 (19%) | 4 (12%) | 7 (27%) |
| SOLAS | 12 (35%) | 14 (50%) | 13 (45%) | 6 (24%) |

Did not use pain relief, n (%)

|  | Time | | | |
| --- | --- | --- | --- | --- |
|  | Baseline | 6 weeks | 2 months | 6 months |
| **Hip** | | | | |
| Usual PT | 1(7%) | 2 (18%) | 1 (8%) | 4 (40%) |
| SOLAS | 3(25%) | 5 (50%) | 5 (50%) | 5 (46%) |
| **Knee** | | | | |
| Usual PT | 3 (12%) | 8 (38%) | 7 (32%) | 9 (47%) |
| SOLAS | 7 (24%) | 9 (35%) | 6 (22%) | 5 (23%) |
| **Back** | | | | |
| Usual PT | 4 (10%) | 7 (23%) | 10 (30%) | 7(27%) |
| SOLAS | 5 (15%) | 9 (32%) | 9 (31%) | 8 (32%) |

Followed healthy eating guidelines, n (%)

|  | Time | | | |
| --- | --- | --- | --- | --- |
|  | Baseline | 6 weeks | 2 months | 6 months |
| **Hip** | | | | |
| Usual PT | 8 (57%) | 9 (82%) | 10 (83%) | 9 (90%) |
| SOLAS | 9 (75%) | 10 (100%) | 10 (100%) | 11 (100%) |
| **Knee** | | | | |
| Usual PT | 13 (50%) | 20 (95%) | 19 (86%) | 18 (95%) |
| SOLAS | 24 (83%) | 26 (100%) | 25 (93%) | 22 (100%) |
| **Back** | | | | |
| Usual PT | 23 (56%) | 28 (90%) | 29 (88%) | 22 (85%) |
| SOLAS | 21 (64%) | 28 (100%) | 27 (93%) | 25 (100%) |

*Secondary Outcomes*

SF-12 PCS, mean (SD)

|  | Time | | |
| --- | --- | --- | --- |
|  | Baseline | 2 months | 6 months |
| **Hip** | | | |
| Usual PT | 40.5 (9.3) | 43.9 (4.9) | 45.0 (8.4) |
| SOLAS | 39.4(10.1) | 45.2 (5.7) | 42.7 (7.9) |
| **Knee** | | | |
| Usual PT | 41.9 (8.8) | 44.5(7.2) | 43.9 (8.0) |
| SOLAS | 39.5(8.5) | 44.3 (8.1) | 39.3 (10.1) |
| **Back** | | | |
| Usual PT | 38.6 (10.6) | 42.8 (9.2) | 43.7 (9.3) |
| SOLAS | 39.8 (8.6) | 43.4 (7.0) | 43.4 (9.5) |

Pain NRS, mean (SD)

|  | Time | | |
| --- | --- | --- | --- |
|  | Baseline | 2 months | 6 months |
| **Hip** | | | |
| Usual PT | 5.6 (1.7) | 4.1 (1.9) | 4.0 (2.5) |
| SOLAS | 5.5 (3.0) | 4.2 (2.2) | 4.6 (2.7) |
| **Knee** | | | |
| Usual PT | 6.3 (2.5) | 4.3 (2.9) | 4.9 (2.8) |
| SOLAS | 5.9 (2.4) | 4.6 (2.1) | 4.2 (2.8) |
| **Back** | | | |
| Usual PT | 6.4 (1.9) | 4.3 (2.1) | 4.6 (2.3) |
| SOLAS | 6.2 (2.9) | 5.0 (2.6) | 4.6 (2.7) |

Pain Bothersomeness, mean (SD)

|  | Time | | |
| --- | --- | --- | --- |
|  | Baseline | 2 months | 6 months |
| **Hip** | | | |
| Usual PT | 3.1 (1.1) | 2.8 (1.1) | 2.6 (1.3) |
| SOLAS | 3.1 (1.3) | 2.5 (0.9) | 2.7 (1.0) |
| **Knee** | | | |
| Usual PT | 2.9 (1.2) | 2.6 (1.2) | 2.6 (1.1) |
| SOLAS | 2.8 (0.9) | 2.7 (0.8) | 2.8 (1.2) |
| **Back** | | | |
| Usual PT | 3.4 (0.9) | 2.6 (0.9) | 2.7 (1.0) |
| SOLAS | 3.2 (1.1) | 2.7 (1.2) | 2.7 (1.2) |

HADS Total score, mean (SD)

|  | Time | | |
| --- | --- | --- | --- |
|  | Baseline | 2 months | 6 months |
| **Hip** | | | |
| Usual PT | 10.5 (7.4) | 8.8 (5.6) | 8.8 (10.9) |
| SOLAS | 11.3 (5.9) | 8.8 (5.1) | 9.70 (6.5) |
| **Knee** | | | |
| Usual PT | 11.5 (6.2) | 8.8 (5.7) | 9.1 (6.3) |
| SOLAS | 10.3 (5.9) | 8.8 (6.4) | 8.2 (5.5) |
| **Back** | | | |
| Usual PT | 13.3 (6.4) | 10.6 (7.3) | 9.0 (7.1) |
| SOLAS | 13.8 (6.4) | 9.8 (8.6) | 8.9(6.6) |

HADS Anxiety scale, mean (SD)

|  | Time | | |
| --- | --- | --- | --- |
|  | Baseline | 2 months | 6 months |
| **Hip** | | | |
| Usual PT | 6.0 (4.2) | 5.5 (4.3) | 5.0 (6.1) |
| SOLAS | 6.6 (3.5) | 4.7(3.1) | 5.0 (3.4) |
| **Knee** | | | |
| Usual PT | 6.9 (4.0) | 5.2 (3.3) | 5.3 (3.0) |
| SOLAS | 6.4 (3.8) | 4.9 (3.7) | 5.0 (3.4) |
| **Back** | | | |
| Usual PT | 7.8 (4.3) | 5.7 (4.2) | 4.5 (3.7) |
| SOLAS | 8.9 (4.2) | 5.7 (4.9) | 5.3 (4.1) |

HADS Depression scale, mean (SD)

|  | Time | | |
| --- | --- | --- | --- |
|  | Baseline | 2 months | 6 months |
| **Hip** | | | |
| Usual PT | 4.5 (4.0) | 3.3 (2.2) | 3.8 (5.1) |
| SOLAS | 4.7 (3.1) | 4.1 (2.3) | 4.7 (3.6) |
| **Knee** | | | |
| Usual PT | 4.6 (3.4) | 3.5 (3.1) | 3.8 (3.8) |
| SOLAS | 3.9 (2.9) | 3.9 (2.9) | 3.2 (2.8) |
| **Back** | | | |
| Usual PT | 5.5 (3.2) | 4.9 (3.6) | 4.5 (3.8) |
| SOLAS | 5.4 (3.2) | 4.2 (3.9) | 3.7 (3.1) |

EQ-5D medians (25^th^, 75^th^ centile)

|  | Time | | |
| --- | --- | --- | --- |
|  | Baseline | 2 months | 6 months |
| **Hip** | | | |
| Usual PT | 0.71 (*0.62, 0.73*) | 0.69 (*0.66, 0.80*) | 0.73 (*0.62, 0.80*) |
| SOLAS | 0.69 (*0.64, 0.78*) | 0.76 (*0.69, 0.76*) | 0.69 (*0.62, 0.80*) |
| **Knee** | | | |
| Usual PT | 0.69 (*0.62, 0.73*) | 0.69 (*0.66, 0.76*) | 0.73 (*0.62, 0.76*) |
| SOLAS | 0.73 (*0.62, 0.80*) | 0.73 (*0.67, 0.80*) | 0.69 (*0.57, 0.80*) |
| **Back** | | | |
| Usual PT | 0.69 (*0.59, 0.73*) | 0.73 (*0.66, 0.80*) | 0.74 (*0.69, 0.80*) |
| SOLAS | 0.69 (*0.59, 0.73*) | 0.69 (*0.62, 0.76*) | 0.71 (*0.62, 0.80*) |

GPE, mean (SD)

|  | Time | | |
| --- | --- | --- | --- |
|  | Baseline | 2 months | 6 months |
| **Hip** | | | |
| Usual PT | -1.6 (2.4) | 2.3 (1.5) | 1.2 (2.7) |
| SOLAS | -1.8 (2.2) | 1.7 (2.5) | 0.9 (2.9) |
| **Knee** | | | |
| Usual PT | -2.3 (2.0) | 2.1 (2.1) | 0.9 (2.2) |
| SOLAS | -1.8 (2.5) | 1.7 (2.8) | 0.6 (2.9) |
| **Back** | | | |
| Usual PT | -1.8 (2.1) | 1.9 (1.3) | 1.3 (2.1) |
| SOLAS | -2.3 (3.0) | 1.5 (2.5) | 1.3 (2.6) |

*Selected Determinants of Self-Management Behaviour for Back Pain participants only*

TSK-11 Activity avoidance subscale, mean (95% CI)

|  | Baseline | Mean diff 6 weeks | Group diff | Mean diff  2 months | Group  diff | Mean diff  6 months | Group diff |
| --- | --- | --- | --- | --- | --- | --- | --- |
| Usual PT  (n=41) | 16.7  (15.6, 17.8) | -1.9  (-2.9,  -0.9) | -1.6  (-3.1, -0.1) | -2.2  (-3.3, -1.2) | -1.0  (-2.6, 0.5) | -1.2  (-2.3, -0.1) | -0.1  (-1.8, 1.5) |
| SOLAS  (n=34) | 15.9 (14.7, 17.1) | -0.3 (-1.4, 0.8) |  | -1.2  (-3.0, -0.1) |  | -1.1  (-2.3, 0.1) |  |

PCS, mean (95% CI)

|  | Baseline | Mean diff 6 weeks | Group diff | Mean diff  2 months | Group  diff | Mean diff  6 months | Group diff |
| --- | --- | --- | --- | --- | --- | --- | --- |
| Usual PT | 19.3  (15.2, 23.3) | -6.7  (-10.1, -3.2) | -0.8  (-5.8, 4.2) | -8.6  (-12.1,  -5.2) | -2.0  (-7.1, 2.9) | -11.1  (-14.7, -7.4) | -1.5  (-6.8, 3.9) |
| SOLAS | 21.3  (16.9, 25.8) | - 5.9  (-9.5, -2.2) |  | -6.6  (-10.3,  -2.9) |  | -9.6  (-13.5,  -5.7) |  |
