# Supplementary material for: Feasibility cluster randomised controlled trial evaluating a theory-driven group-based complex intervention versus usual physiotherapy to support self-management of osteoarthritis and low back pain (SOLAS)
Source: Trials. 2020 Sep 23;21:807. doi: 10.1186/s13063-020-04671-x (PMC7510107; doi:10.1186/s13063-020-04671-x)
Supplement: Supplementary file 13 — Additional file 13. CONSORT Checklist. [file 13063_2020_4671_MOESM13_ESM.docx]

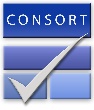
CONSORT 2010 checklist of information to include when reporting a pilot or feasibility trial* and when reporting a cluster randomised trial

| Section/Topic | Item No | Checklist item | Reported on page No |
| --- | --- | --- | --- |
| Title and abstract | | | |
|  | 1a | Identification as a pilot or feasibility randomised trial and cluster trial in the title | 1 |
|  | 1b | Structured summary of pilot trial design, methods, results, and conclusions (for specific guidance see CONSORT abstract extension for pilot trials) | 3-4 |
| Introduction | | | |
| Background and objectives | 2a | Scientific background and explanation of rationale for future definitive trial, and reasons for randomised pilot trial and cluster trial | 5-6 |
|  | 2b | Specific objectives or research questions for pilot trial and whether objectives pertain to the cluster level, the individual participant or both | 6-7 |
| Methods | | | |
| Trial design | 3a | Description of pilot trial design (such as parallel, factorial) including allocation ratio and definition of cluster and description of how the design features apply to the clusters | 7-8 |
|  | 3b | Important changes to methods after pilot trial commencement (such as eligibility criteria), with reasons | N/A |
| Participants | 4a | Eligibility criteria for participants and clusters | 7-9, Table 1 |
|  | 4b | Settings and locations where the data were collected | 7 |
|  | 4c | How participants were identified and consented | 7-9 |
| Interventions | 5 | The interventions for each group with sufficient details to allow replication, including how and when they were actually administered and whether interventions pertain to the cluster level, the individual participant level or both | 9-11 |
| Outcomes | 6a | Completely defined prespecified assessments or measurements to address each pilot trial objective specified in 2b, including how and when they were assessed and whether the outcome measures pertain to the cluster level, the individual participant level or both | 11 |
|  | 6b | Any changes to pilot trial assessments or measurements after the pilot trial commenced, with reasons | N/A |
|  | 6c | If applicable, prespecified criteria used to judge whether, or how, to proceed with future definitive trial | 12 |
| Sample size | 7a | Rationale for numbers in the pilot trial, and method of calculation, number of clusters and whether equal or unequal cluster sizes are assumed, cluster size, a coefficient of intracluster correlation (ICC or *k*) and an indication of uncertainty | 12 |
|  | 7b | When applicable, explanation of any interim analyses and stopping guidelines | 13 |
| Randomisation: |  |  |  |
| Sequence  generation | 8a | Method used to generate the random allocation sequence | 8 – clusters |
|  | 8b | Type of randomisation(s); details of any restriction (such as blocking and block size), details of stratification or matching if used | 7-8– cluster |
| Allocation  concealment  mechanism | 9 | Mechanism used to implement the random allocation sequence (such as sequentially numbered containers), describing any steps taken to conceal the sequence until interventions were assigned, specification that allocation was based on clusters rather than individuals and whether allocation concealment (if any) was at cluster level, the individual participant level or both | 8 |
| Implementation | 10a | Who generated the random allocation sequence, who enrolled clusters, and who assigned clusters to interventions | 8 |
|  | 10b | Mechanism by which individual participants were included in clusters for the purposes of the trial (such as complete enumeration, random sampling) | 8-9 |
|  | 10c | From whom consent was sought (representatives of the cluster, or individual cluster members, or both) and whether consent was sought before or after randomisation | 7-9 |
| Blinding | 11a | If done, who was blinded after assignment to interventions (for example, participants, care providers, those assessing outcomes) and how | 8-9, Additional files 1, 2 |
|  | 11b | If relevant, description of the similarity of interventions | 9 |
| Statistical methods | 12 | Methods used to address each pilot trial objective whether qualitative or quantitative, and how clustering was taken into account | 13-14 |
| Results | | | |
| Participant flow (a diagram is strongly recommended) | 13a | For each group, the numbers of clusters who were approached and/or assessed for eligibility, randomly assigned, received intended treatment, and were assessed for each objective | 14 |
|  | 13b | For each group, losses and exclusions after randomisation for both clusters and individual cluster members, together with reasons | 14-15 |
| Recruitment | 14a | Dates defining the periods of recruitment and follow-up | 13-15 |
|  | 14b | Why the pilot trial ended or was stopped | N/A |
| Baseline data | 15 | A table showing baseline demographic and clinical characteristics for the individual and cluster levels as applicable for each group | Additional file 9 |
| Numbers analysed | 16 | For each objective, number of clusters and participants (denominator) included in each analysis. If relevant, these numbers should be by randomised group | Figure 2 |
| Outcomes and estimation | 17 | For each objective, results including expressions of uncertainty (such as 95% confidence interval) for any  Estimates and results at the individual or cluster level as applicable and a coefficient of intracluster correlation (ICC or *k*) for each primary outcome. If relevant, these results should be by randomised group | Tables 2-6, Additional files 10-12, pages 14-20 |
| Ancillary analyses | 18 | Results of any other analyses performed that could be used to inform the future definitive trial | 19, Additional file 12 |
| Harms | 19 | All important harms or unintended effects in each group (for specific guidance see CONSORT for harms) | 10, 11, and 15 |
|  | 19a | If relevant, other important unintended consequences | N/A |
| Discussion | | | |
| Limitations | 20 | Pilot trial limitations, addressing sources of potential bias and remaining uncertainty about feasibility | 21, 23-24, 27 |
| Generalisability | 21 | Generalisability (applicability) of pilot trial methods and findings to future definitive trial and other studies, and generalisability to clusters and/or individual participants (as relevant) | 21-25 |
| Interpretation | 22 | Interpretation consistent with pilot trial objectives and findings, balancing potential benefits and harms, and  considering other relevant evidence | 21-28 |
|  | 22a | Implications for progression from pilot to future definitive trial, including any proposed amendments | 23-27 |
| Other information | | |  |
| Registration | 23 | Registration number for pilot trial and name of trial registry | 4 |
| Protocol | 24 | Where the pilot trial protocol can be accessed, if available | 4, ref [1] |
| Funding | 25 | Sources of funding and other support (such as supply of drugs), role of funders | 31 |
|  | 26 | Ethical approval or approval by research review committee, confirmed with reference number | 7 |

Citation: Eldridge SM, Chan CL, Campbell MJ, Bond CM, Hopewell S, Thabane L, et al. CONSORT 2010 statement: extension to randomised pilot and feasibility trials. BMJ. 2016;355.

*We strongly recommend reading this statement in conjunction with the CONSORT 2010, extension to randomised pilot and feasibility trials, Explanation and Elaboration for important clarifications on all the items. If relevant, we also recommend reading CONSORT extensions for cluster randomised trials, non-inferiority and equivalence trials, non-pharmacological treatments, herbal interventions, and pragmatic trials. Additional extensions are forthcoming: for those and for up to date references relevant to this checklist, see [www.consort-statement.org](http://www.consort-statement.org).
